# The ERM-1 membrane-binding domain directs *erm-1* mRNA localization to the plasma membrane in the *C. elegans* embryo

**DOI:** 10.1242/dev.200930

**Published:** 2022-11-21

**Authors:** Lindsay P. Winkenbach, Dylan M. Parker, Robert T. P. Williams, Erin Osborne Nishimura

**Affiliations:** ^1^ Department of Biochemistry and Molecular Biology, Colorado State University, Fort Collins, CO 80523, USA; ^2^ Department of Chemistry and Biochemistry, Howard Hughes Medical Institute, University of Colorado, Boulder, CO 80303, USA

**Keywords:** mRNA localization, *C. elegans*, *erm-1*, FERM domain, Plasma membrane, Translation

## Abstract

mRNA localization and transport are integral in regulating gene expression. In *Caenorhabditis elegans* embryos, the maternally inherited mRNA *erm-1* (*Ezrin/Radixin/Moesin*) becomes concentrated in anterior blastomeres. *erm-1* mRNA localizes within those blastomeres to the plasma membrane where the essential ERM-1 protein, a membrane-actin linker, is also found. We demonstrate that the localization of *erm-1* mRNA to the plasma membrane is translation dependent and requires its encoded N-terminal, membrane-binding (FERM) domain. By perturbing translation through multiple methods, we found that *erm-1* mRNA localization at the plasma membrane persisted only if the nascent peptide remained in complex with the translating mRNA. Indeed, re-coding the *erm-1* mRNA coding sequence while preserving the encoded amino acid sequence did not disrupt *erm-1* mRNA localization, corroborating that the information directing mRNA localization resides within its membrane-binding protein domain. A single-molecule inexpensive fluorescence *in situ* hybridization screen of 17 genes encoding similar membrane-binding domains identified three plasma membrane-localized mRNAs in the early embryo. Ten additional transcripts showed potential membrane localization later in development. These findings point to a translation-dependent pathway for localization of mRNAs encoding membrane-associated proteins.

## Teaser

**Summary:** In *C. elegans*, *erm-1* mRNA localization to plasma membranes requires the ERM-1 membrane-binding domain.

## INTRODUCTION

mRNA localization is a prevalent feature in diverse cell types and organisms (Chouaib et al., 2020; Knowles et al., 1996; Lécuyer et al., 2007; Llopis et al., 2010; Long et al., 1997; Rebagliati et al., 1985). Subcellular localization of mRNA is associated with spatiotemporal control of gene expression. mRNA localization can occur as a cause or consequence of translational regulatory control, and can promote mRNA degradation, facilitate interactions with effector proteins and prevent premature non-specific interactions (Besse and Ephrussi, 2008; Broadus et al., 1998; Chouaib et al., 2020; Ephrussi et al., 1991; Frohnhöfer and Nüsslein-Volhard, 1986; Ma and Mayr, 2018; Rebagliati et al., 1985; Ryder and Lerit, 2018; Sepulveda et al., 2018). In *Caenorhabditis elegans* early embryos, mRNA localization is a prominent feature of maternally inherited mRNAs and may contribute to cell-specific patterning prior to the onset of zygotic transcription (Aoki et al., 2021; Lee et al., 2020; Nishimura et al., 2015; Parker et al., 2020; Updike and Strome, 2010). Generally, maternal transcripts enriched in the posterior cells of the embryo localize to membraneless biomolecular condensates called P granules (Lee et al., 2020; Parker et al., 2020). Previous work has indicated that transcripts localize to P granules following translation repression (Lee et al., 2020; Parker et al., 2020). In contrast, maternal transcripts that concentrate in the anterior cells often localize to the plasma membrane (Parker et al., 2020), frequently colocalizing with their encoded proteins. However, the molecular mechanisms that facilitate membrane localization in *C. elegans* are unclear. Here, we focus on *erm-1* as a model membrane-associated transcript and characterize the mechanisms underlying its localization to the cell membrane.

mRNA localization can occur via translation-independent or translation-dependent pathways (St Johnston, 2005; Parton et al., 2014; Szostak and Gebauer, 2013). Translation-independent pathways typically rely on *cis-*acting elements, RNA sequences or structures that are often located in untranslated regions (UTRs) and recruit *trans-*recognition factors, such as RNA-binding proteins (RBPs). Recognition by RBPs can lead to either passive or directed transport, often in association with other processes, such as mRNA protection, mRNA degradation, or translational regulatory control (Engel et al., 2020). The result is an enrichment of mRNAs in specific subcellular locales. In *C. elegans*, some posterior-enriched maternal transcripts localize through translation-independent pathways, relying on *cis*-acting elements in their 3′UTRs to direct translational repression, which is required for localization into P granules (Lee et al., 2020; Parker et al., 2020). In contrast, translation-dependent pathways of mRNA localization typically rely on peptide signals in the nascent polypeptide. Transcripts that concentrate at the endoplasmic reticulum (ER) often rely on signal peptides to direct translating mRNAs and their encoded proteins to their destinations (Walter and Johnson, 1994). However, previously identified transcripts that localize to the plasma membrane in *C. elegans* embryos lack a discernable signal peptide (Parker et al., 2020). Recently, several *C. elegans* transcripts that encode members of the apical junction sub-complexes, their additional ancillary proteins or other cytoskeletal components were found to localize to the plasma membrane during mid-embryogenesis with subcellular localization patterns that did not appear to overlap with the ER (Li et al., 2021; Tocchini et al., 2021). Of these, the *dlg-1* transcript was shown to localize in a translation-dependent fashion (Tocchini et al., 2021). Together, these findings demonstrate that the localization of plasma membrane-enriched transcripts can occur in a manner distinct from the canonical, ER signal peptide-directed pathway and that local translation may be a general feature of junction and membrane-linker proteins.

*erm-1* (*ezrin/radixin/moesin*) mRNA is the most anterior-enriched transcript in the two-cell *C. elegans* embryo (Nishimura et al., 2015; Tintori et al., 2016). In addition to its high enrichment within anterior cells of the early embryo, *erm-1* mRNA becomes concentrated at plasma membranes within those cells, a pattern coincident with its encoded ERM-1 protein (van Furden et al., 2004; Göbel et al., 2004; Parker et al., 2020). Previously, we showed that the *erm-1* 3′UTR was insufficient to direct membrane mRNA localization, indicating that the localization element resides elsewhere in the RNA sequence or the encoded protein (Parker et al., 2020). In this study, we set out to identify which elements in *erm-1* mRNA or the encoded ERM-1 protein are necessary for membrane localization.

In *C. elegans*, ERM-1 is the sole ortholog of the conserved ERM protein family, members of which serve as membrane-actin linkers (van Furden et al., 2004). ERM proteins regulate cell morphology and signaling events at the plasma membrane. Therefore, they are prominent in processes such as epithelial junction remodeling, cell migration, promotion of microvilli formation, and interactions with actin at the cell cortex (Fehon et al., 2010; van Furden et al., 2004; Göbel et al., 2004; McClatchey, 2014). Proper specialization of the cell cortex and plasma membrane is crucial for controlling cell morphology, as evidenced by the fact that in *C. elegans* loss of *erm-1* in the intestine results in early embryo lethality due to constrictions and disjunctions in the intestinal lumen (van Furden et al., 2004; Ramalho et al., 2020).

Here, we demonstrate that *erm-1* mRNA accumulation at the plasma membrane is translation dependent and requires the membrane-binding ability of the FERM domain to become enriched at the plasma membrane. Furthermore, we screened 17 genes encoding similar membrane-binding FERM or PH-like domains. We identified 12 additional transcripts with plasma membrane or other patterns of subcellular mRNA localization that change over developmental time. Our findings suggest that translation of this conserved membrane-binding domain is conducive to subcellular localization of both the mRNA and the encoded protein.

## RESULTS

### *erm-1* mRNA localization to the plasma membrane requires translation initiation

**Fig. 1.**
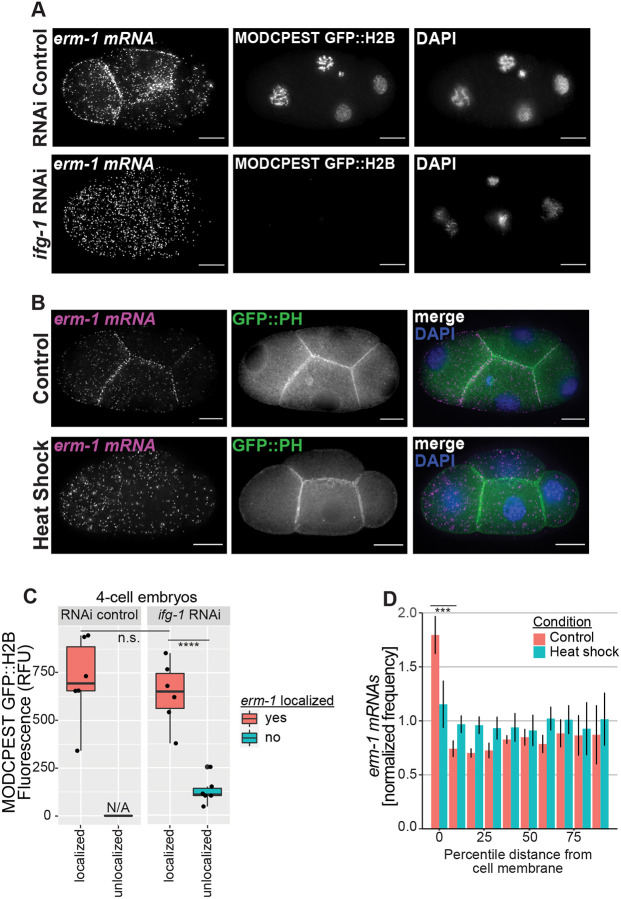
**Disruption of global translation leads to loss of *erm-1* mRNA localized to the membrane.** (A) Fluorescence micrographs of four-cell-stage *C. elegans* embryos. RNAi was performed to deplete the translational initiation factor *ifg-1*, or an empty vector RNAi control was performed. *erm-1* transcripts were imaged by smFISH. In the same embryos, GFP from a translational reporter transgene (MODCPEST-GFP::H2B) and DNA (DAPI) were also imaged. Representative images are shown from a total of 102 four-cell-stage embryos surveyed [*n*=23 RNAi control, *n*=79 *ifg-1* RNAi (*n*=43 reporter signal retained, *n*=36 reporter signal depleted)]. Scale bars: 10 µm. (B) Four-cell-stage *C. elegans* embryos harboring a membrane marker transgene (GFP::PH, green) were imaged for *erm-1* transcripts by smFISH (magenta) under no heat shock (control 20°C) and under heat stress conditions (25 min at 30°C heat shock). DNA was also imaged as DAPI staining (blue). Scale bars: 10 µm. (C) Quantification of translation reporter fluorescence under RNAi control (*n=*6), *ifg-1* RNAi *erm-1* localization retained (*n*=6) and *ifg-1* RNAi *erm-1* localization lost (*n*=6) conditions. Background-subtracted GFP intensities were measured as relative fluorescence units (RFU) using nuclear masks generated from DAPI staining. *erm-1* mRNA localization was assessed qualitatively as localized or unlocalized in four-cell embryos. ****P<0.00005 (Welch two-sample *t*-tests comparing RFU values for localized versus unlocalized for the transcript *erm-1* at the given condition). n.s., not significant (*P*>0.05). (D) Quantification of *erm-1* mRNA under control (a representative set of *n*=5) and heat-shock conditions (a representative set of *n*=7) indicating the normalized frequency of *erm-1* mRNA at increasing, normalized distances from the cell periphery. ***P<0.0005 (Welch two-sample *t*-tests comparing the cell membrane localization for heat shock versus control for the transcript *erm-1* at the given stage). Error bars represent s.d.

mRNA localization is directed through either translation-dependent or translation-independent pathways (St Johnston, 2005; Parton et al., 2014; Szostak and Gebauer, 2013). To test which pathway was responsible for *erm-1* mRNA localization, we disrupted global translation by two methods and determined whether either perturbed *erm-1* mRNA accumulation at the membrane. We first depleted the translation initiation factor *ifg-1* [*Initiation Factor 4G (eIF4G) family*] by RNA interference (RNAi). IFG-1 is the sole *C. elegans* ortholog of eIF4G, and both cap-dependent and -independent translation initiation require IFG-1 (Berset et al., 1998; Kim et al., 2018; Ramírez-Valle et al., 2008; Rogers et al., 2011). Using a destabilized-GFP as a translation reporter (MODCPEST GFP::H2B), we found that *ifg-1* RNAi decreased translation in a partially penetrant fashion as indicated by a decrease in GFP::H2B fluorescence (Corish and Tyler-Smith, 1999; Kaymak et al., 2016; Li et al., 1998) (Fig. 1A,C). The destabilized MODCPEST GFP::H2B was constructed from the protein degradation-inducing PEST sequence and the degradation domain of mouse ornithine decarboxylase (MODC) and has a half-life of roughly 2 h (compared with 26 h for unmodified GFP::H2B) (Corish and Tyler-Smith, 1999; Kaymak et al., 2016; Li et al., 1998). *ifg-1* RNAi introduced in the L2 stage of development led to 46% of four-cell progeny exhibiting a significant loss of GFP signal and 54% showing no significant change compared with wild type. Importantly, we observed that embryos with significantly reduced MODCPEST GFP::H2B also experienced a qualitative loss of *erm-1* mRNA localization at the plasma membrane with high concordance (Fig. 1A,C). These results support the model that *erm-1* mRNA localization to the plasma membrane correlates with translation.

As a complementary approach to disrupting global translation initiation via RNAi, we next disrupted translation through heat shock and quantified the resulting changes in *erm-1* mRNA enrichment at the plasma membrane (Parker et al., 2020). Heat shock prevents protein synthesis primarily through changes in phosphorylation states of translation initiation factors followed by their subsequent inactivation (Cuesta et al., 2000; Duncan and Hershey, 1984; Shalgi et al., 2013). Heat shock acts within a shorter time frame than *ifg-1* RNAi (25 min heat shock versus 48 h RNAi exposure). In heat-treated four-cell embryos, we observed a 37% reduction in *erm-1* mRNA enrichment alongside the plasma membrane (at a distance within 10% of the normalized radius from the plasma membrane) after 25 min at 30°C compared with 20°C controls (Fig. 1B,D). Combined with the *ifg-1* RNAi experiment findings, this illustrated that *erm-1* mRNA localization to the plasma membrane depends on translation initiation for establishment. An advantage of using four-cell early embryos is that zygotic transcription has not yet initiated, and therefore any observed results arise from more direct, post-transcriptional processes. Together, these results suggest a translation-dependent pathway and imply that the signal to localize *erm-1* mRNA to membranes may be an encoded peptide sequence in the nascent ERM-1 protein. However, these assays do not yield information on whether active translation or an intact ribosome nascent chain complex (RNC), or both, are required for localization.

### The ERM-1 nascent peptide is required for *erm-1* mRNA enrichment at plasma membranes

We identified that translation is required for *erm-1* mRNA enrichment at plasma membranes. This suggests a model in which the RNC – comprising *erm-1* mRNA, the translating ribosome and the emerging nascent ERM-1 protein – is transported to the plasma membrane together through recognition of amino acid sequences in the nascent ERM-1 protein. We hypothesize that *erm-1* mRNA localization requires intact RNCs, likely at steps that both establish and maintain localization. To test this hypothesis, we inhibited translation elongation using two different drugs, one that preserves RNCs (cycloheximide) and one that disrupts them (puromycin) (Azzam and Algranati, 1973; Schneider-Poetsch et al., 2010).

The eggshell and permeability barrier in the *C. elegans* embryo complicate drug treatment by limiting small molecule penetrance (Carvalho et al., 2011; Olson et al., 2012; Stein and Golden, 2018). To circumvent this, we disrupted the sugar-modifying enzyme and permeability barrier protein PERM-1 by RNAi, thereby allowing ingression of small molecules such as cycloheximide and puromycin. Although *perm-1* RNAi eventually leads to lethality in late embryos, development in early embryonic stages proceeds normally (Carvalho et al., 2011). Importantly, *perm-1* RNAi is compatible with both drug treatment and single-molecule fluorescent *in situ* hybridization (smFISH) imaging of *erm-1* mRNA.

**Fig. 2.**
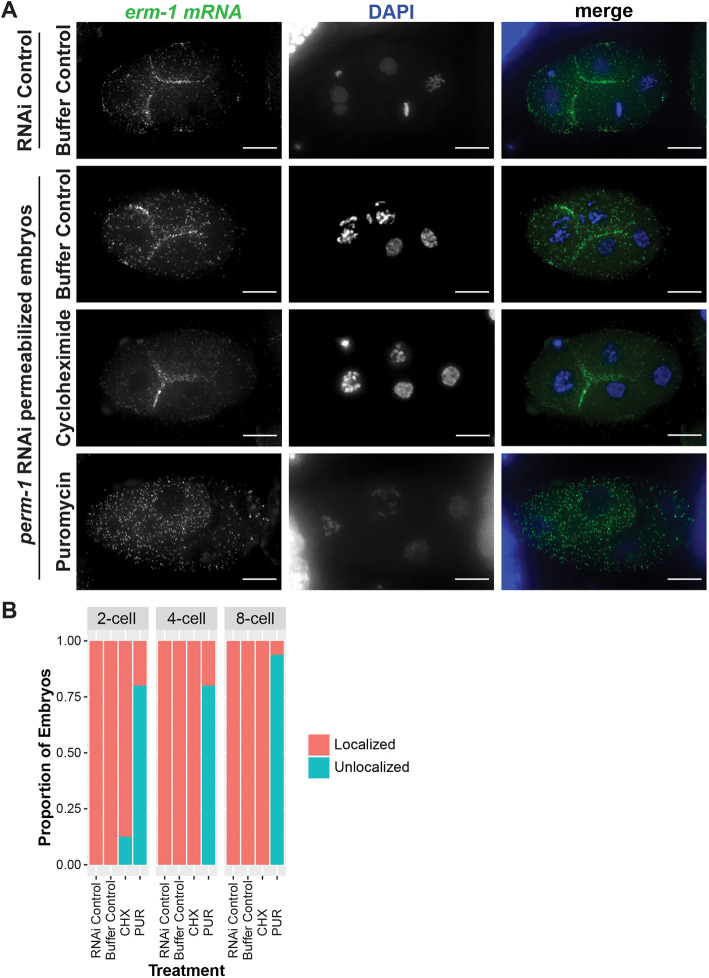
**The intact ERM-1 RNC is required for *erm-1* mRNA enrichment at cell membranes.** (A) Fluorescence micrographs of four-cell-stage *C. elegans* embryos are shown in which embryos were permeabilized by *perm-1* RNAi and subsequently treated with small molecule translation inhibitors, in comparison to RNAi and drug treatment controls. *erm-1* mRNA (green) was imaged by smFISH, under control, cycloheximide (CHX; 500 µg/ml, 20 min) or puromycin (PUR; 500 µg/ml, 20 min) treatment conditions (*n*=9 RNAi control, *n*=13 buffer control, *n*=4 CHX, *n*=25 PUR). Scale bars: 10 µm. (B) Bar plot indicating the proportion of embryos displaying *erm-1* mRNA enriched at cell membranes (localized) or homogenously distributed through the cell (unlocalized) for two-cell, four-cell and eight-cell embryos subjected to the indicated treatments (two-cell: *n*=10 RNAi control, *n*=8 buffer control, *n*=8 CHX, *n*=20 PUR; 8-cell: *n*=10 RNAi control, *n*=4 buffer control, *n*=10 CHX, *n*=16 PUR).

We observed that disruption of the RNC by puromycin treatment led to loss of *erm-1* mRNA localization at the membrane in 84% of embryos between the two-cell and eight-cell stages (Fig. 2; Fig. S1). In contrast, cycloheximide treatment, which stalls translation during elongation while preserving the RNC only altered *erm-1* mRNA localization in 4% of embryos surveyed (Fig. 2; Fig. S1). This suggests that the *erm-1* mRNA must remain associated with the ribosome for *erm-1* mRNA molecules to maintain localization to plasma membranes upon translation disruption. Additionally, the maintenance of *erm-1* mRNA localization does not require ongoing translational elongation provided stalled RNCs are preserved intact, as is the case with cycloheximide treatment. These findings further support the translation-dependent model and suggest that *erm-1* mRNA transcripts localize through association with the RNC.

### *erm-1* mRNA localization to the plasma membrane does not depend on nucleic acid sequences

**Fig. 3.**
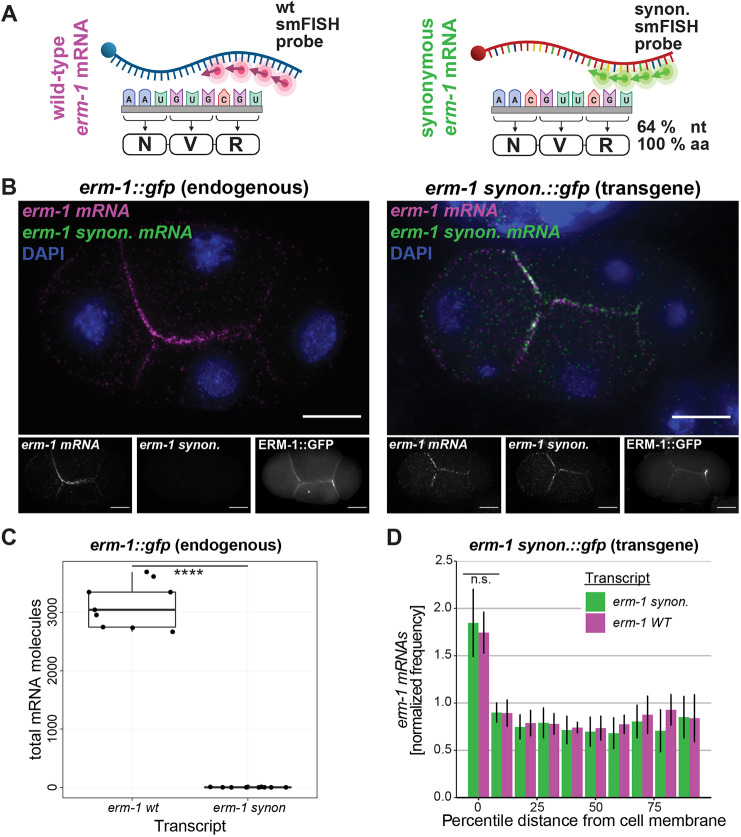
***erm-1* mRNA localization to the cell membrane is mRNA coding sequence independent.** (A) Schematics comparing wild-type *erm-1* mRNA (magenta) with the re-coded, synonymous *erm-1* mRNA (green). (B) smFISH micrographs of four-cell-stage *C. elegans* embryos imaging wild-type *erm-1* (magenta) and the re-coded, synonymous *erm-1* mRNA (*erm-1* synon. mRNA, green). GFP-tagged *erm-1* control four-cell embryo (left) and MosSCI GFP-tagged synonymous *erm-1* four-cell embryo (right) are shown with DNA marked by DAPI (blue) and membranes marked by ERM-1::GFP. Scale bars: 10 µm. (C) Total number of *erm-1 wt* and *erm-1* synonymous mRNA molecules detected in *erm-1*::*gfp* (endogenous) (*n*=9). ****P<0.00005 (Welch two-sample *t*-tests comparing the total number of *erm-1* molecules detected by the *erm-1 wt* probe versus the *erm-1* synonymous probe in the endogenously tagged *erm-1::gfp* background)*.* (D) Quantification of endogenous and synonymous *erm-1* mRNA (*n*=6) indicating the normalized frequency of mRNAs within binned, percentile distances from the cell membrane counted and normalized against the total volume of each cell. n.s., not significant (*P*>0.05; Welch two-sample *t*-tests comparing the cell membrane localization of endogenous and synonymous *erm-1* mRNA in the *erm-1 synon::gfp* transgenic background). Error bars represent s.d.

We have established that *erm-1* mRNA localizes to plasma membranes in a translation-dependent manner. However, *erm-1* mRNA can persist at membranes if the RNC remains intact when disrupting translation elongation. This suggests that localization is dependent on the ERM-1 amino-acid sequence and not the *erm-1* mRNA sequence. Supporting this hypothesis, previous evidence has illustrated that the *erm-1* 3′UTR (typically a common site of *cis*-acting localization elements) is insufficient to direct mRNA to the membrane (Parker et al., 2020). To test whether other *erm-1* mRNA nucleotide sequences are dispensable for localization, we artificially re-coded the *erm-1* mRNA nucleotide sequence while preserving the amino acid sequence by capitalizing on the redundancy of the genetic code (Fig. S2A). Our nucleotide re-coded, yet amino-acid synonymous, *erm-1* sequence (called *erm-1 synon*) shares 64% identity at the nucleic acid level with the wild-type *erm-1* sequence (called *erm-1*) while maintaining 100% identity of the amino acid sequence (Fig. 3A; Fig. S2B). We designed single-molecule inexpensive FISH (smiFISH) probes that could distinguish between the re-coded, synonymous *erm-1* and wild-type sequences (Fig. 3B,C). Using these probes, we found that the *erm-1 synon* transcript retained enrichment at the plasma membrane with no significant difference between it and either the endogenous *erm-1* transcript (Fig. 3B,D) or a matched transgenic wild-type *erm-1* sequence inserted at the same transgenic location (Fig. S2C,D). These data imply that RNA sequences within the *erm-1* transcript are dispensable for *erm-1* mRNA localization, and, instead, localization elements reside in the translated ERM-1 protein.

### *erm-1* mRNA and ERM-1 protein localization require the ERM-1 PIP_2_ membrane-binding region

To identify domains within the ERM-1 protein required to localize translating *erm-1* to plasma membranes, we examined *erm-1* mRNA localization upon mutating key conserved ERM-1 domains. Generally, ERM proteins are a conserved family defined by domains common to their founding members: Ezrin, Radixin and Moesin (Bretscher, 1983; Lankes and Furthmayr, 1991; Tsukita et al., 1989). ERM proteins serve as structural linkers between the plasma membrane and the actin cytoskeleton and play central roles in cell morphology and signaling processes that converge on the plasma membrane. Two key domains coordinate their linker function. The N-terminal band 4.1 Ezrin/Radixin/Moesin (FERM) domain houses a PH-like (Pleckstrin homology-like) domain that associates with the plasma membrane through interactions with PIP_2_ [phosphatidylinositol (4,5) bisphosphate] (Barret et al., 2000; Fehon et al., 2010; Roch et al., 2010). In contrast, the C-terminal ERM association domain (C-ERMAD) interacts with the actin cytoskeleton in a phosphorylation-dependent manner (McClatchey, 2014; Ramalho et al., 2020). The FERM and C-ERMAD domains can also intramolecularly bind to prevent their respective substrate associations. A dephosphorylation event on C-ERMAD increases intramolecular affinity, switching the protein into the inactive form (Li et al., 2007; Pearson et al., 2000; Roch et al., 2010). Therefore, the architecture of ERM-1 connects the plasma membrane and the actin cytoskeleton via a phosphorylation-dependent mechanism.

**Fig. 4.**
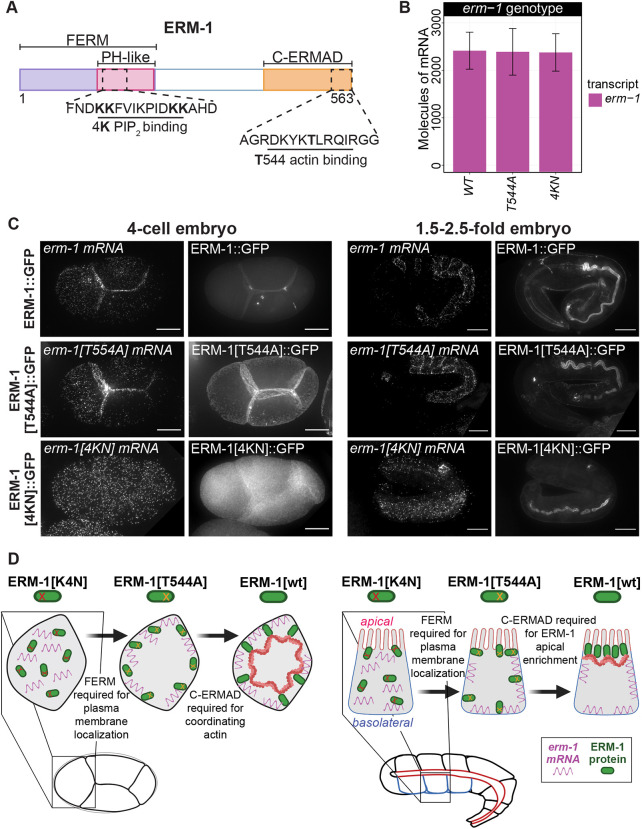
**PIP_2_ binding is required for *erm-1* mRNA and ERM-1 protein localization in early and late embryos.** (A) Schematic of the ERM-1 protein showing the N-terminal FERM domain (purple) containing the conserved PIP_2_ membrane-binding region (PH-like, pink) and C-terminal conserved C-ERMAD actin-binding domain (orange). (B) *erm-1* mRNA abundance does not significantly differ between *erm-1::gfp* (WT), *erm-1[T544A]::gfp* and *erm-1[4KN]::GFP* strains. (*n*=10 *erm-1*, *n*=10 *erm-1[T544A]*, *n*=9 *erm-1[4KN]*)*. P*>0.05 (Welch two-sample *t*-tests comparing the number of *erm-1* mRNA molecules detected in *erm-1::gfp*, *erm-1[T544A]::gfp* and *erm-1[4KN]::GFP* strains). Error bars represent s.d. (C) smFISH micrographs of four-cell and 2.5-fold embryos displaying *erm-1* mRNA and ERM-1 protein localization in *erm-1::gfp*, *erm-1[T544A]::gfp* and *erm-1[4KN]::GFP* strains. Scale bars: 10 µm. (D) Depiction of *erm-1* mRNA and ERM-1 protein localization at in four-cell-stage and mid-stage embryos illustrating the contributions of the FERM and T544 regions. In polarized intestinal cells, ERM-1 localizes in a two-step process. Created using BioRender.com

Mutating four lysines to asparagines abrogates the PIP_2_ binding ability of the FERM domain (Barret et al., 2000; Roch et al., 2010) (Fig. 4A), termed the ERM-1[4KN] mutant (Ramalho et al., 2020). In *C. elegans*, this leads to intestinal lumen cysts and disjunctions as well as early larval lethality that phenocopy *erm-1* null mutants (Göbel et al., 2004; Ramalho et al., 2020). In contrast, mutating the conserved, phosphorylatable residue T544 to alanine (ERM-1[T544A]) disrupts the function of C-ERMAD, thereby rendering C-ERMAD non-phosphorylatable (Carreno et al., 2008; Zhang et al., 2020).

In *C. elegans*, the ERM-1 protein localizes to the plasma membrane in blastomeres. However, in intestinal cells, which become polarized in mid-embryonic stages, ERM-1 localization is more complex and is hypothesized to occur in a two-step fashion (Ramalho et al., 2020). In those cells, ERM-1 protein localizes first to the plasma membrane generally and then later concentrates almost exclusively at the apical side, which forms the intestinal lumen. The FERM domain is required or important for localization of ERM-1 protein to the plasma membrane of many cells. This is evidenced by the fact that the ERM-1[K4N]::GFP mutant proteins fail to localize to the plasma membranes of primordial germ cells and seam cells, and are reduced in their localization to apical intestinal cells. Conversely, within polarized intestinal cells, phosphorylation and de-phosphorylation cycling at the T544 residue is hypothesized to direct ERM-1 proteins to relocalize to apical membranes. This is evidenced by the fact that ERM-1[T544A]::GFP and ERM-1[T544D]::GFP mutants both experience delays or reductions in apical enrichment of ERM-1 proteins. Loss of ERM-1 at the apical face of intestinal cells leads to disrupted cortical actin organization and lumenal defects (Ramalho et al., 2020). We assessed *erm-1* mRNA localization in these two previously characterized mutant strains at two different stages of development to determine whether *erm-1* mRNA accumulation at the plasma membranes of early-stage cells requires the FERM or C-ERMAD domains. We also assessed how *erm-1* mRNA localization behaves in polarized intestinal cells.

We examined *erm-1* mRNA localization in ERM-1[T544A] homozygous mutants with no wild-type ERM-1 in the background. In four-cell-stage embryos, both *erm-1[T544A]* mRNA and ERM-1[T544A] protein localized to the plasma membranes (Fig. 4C). However, the mutants exhibited GFP localization that appeared ‘ruffled’ or distorted compared with wild-type ERM-1::GFP fusions, indicating that loss of this phospho-moiety imparts a phenotype (Fig. 4C) (Ramalho et al., 2020). Ramalho et al. observed that ERM-1[T544A] mutant protein persisted at basolateral surfaces of intestinal cells until the 2.0-fold stage when all wild-type ERM-1 protein had localized to the apical face (Ramalho et al., 2020). We also observed in 1.5- to 2.5-fold embryos that the ERM-1[T544A] protein persisted basolaterally (Fig. 4C) (Ramalho et al., 2020). However, both *erm-1[T544A]* mRNA and wild-type *erm-1* mRNA failed to concentrate at the apical membrane. Together, this suggests that in polarized cells the T544-dependent movement of ERM-1 protein from the basolateral to apical membranes occurs post-translationally, leaving the *erm-1* mRNA behind (Fig. 4D). Therefore, T544A mutation disrupted ERM-1 protein localization to the apical membranes of polarized cells, but did not disrupt *erm-1* mRNA membrane localization either pre- or post-polarization.

Mutations in ERM-1 that disrupt FERM domain/PIP_2_ interactions (called ERM-1[4KN]) reduce (in the intestine and excretory canal) or eliminate (in progenitor germ cells and seam cells) ERM-1[4KN] protein localization to apical membranes (Ramalho et al., 2020). We performed smFISH on four-cell and mid-stage *erm-1[4KN]/erm-1[4KN]* homozygous mutant embryos (at the endogenous locus) lacking any *erm-1* wild-type copies to assess how this mutation impacts *erm-1* mRNA localization (Fig. 4A; Fig. S3). Homozygosity was determined by assessing *gfp* and *erm-1* mRNA levels. Wild-type *erm-1* homozygotes had no detectable *gfp* mRNA and heterozygotes had detectable *gfp* mRNA levels roughly half of *erm-1* mRNA (Fig. S3)*.* At the four-cell stage, *erm-1[4KN]* mRNA failed to localize to the plasma membrane in all of the nine embryos surveyed (Fig. 4C). ERM-1[4KN] protein enrichment at the plasma membrane was also abolished. The total number of *erm-1[4KN]* mRNA molecules in homozygous mutants was unchanged from *erm-1* mRNA numbers in wild-type embryos at the same stage, indicating that RNA expression or stability was unaffected (Fig. 4B)*.* Membrane localization could not be quantified as GFP was used to report ERM-1 expression and therefore complicated its use as a membrane marker. Similar to its phenotype in early embryos, in polarized intestinal cells at the 1.5-fold stage RNA localization of *erm-1[4KN]* mRNA as well as ERM-1[4KN] protein localization were both disrupted and embryos displayed disjunctions in the lumen, as previously reported (Ramalho et al., 2020). Thus, the FERM domain was required to localize both the *erm-1* mRNA and the ERM-1 protein to the plasma membrane in both polarized and non-polarized cells (Fig. 4D). Combined with our previous findings, this indicates that the peptide signal required to localize the ERM-1 RNC, including its associated *erm-1* mRNA, resides within the FERM domain of the nascent peptide.

### Transcripts encoding FERM and PH-like domains are conducive for localization at the plasma membrane

Given that PIP_2_-binding FERM domains have a high affinity for membranes (Senju et al., 2017), their ability to direct membrane-localized mRNA transcripts may be generalizable. Evidence for this exists in other systems. In early *Drosophila* embryogenesis, the PIP_2_-binding PH and actin-binding domains of the Anillin protein (also known as Scraps) are required to localize *anillin* mRNA to pseudocleavage furrow membranes (Hirashima et al., 2018). Based on the findings from the ERM-1[4KN]::GFP mutant strain, we hypothesized that the PIP_2_-binding element of the FERM domain, the PH-like region, could be a general predictor of transcripts that enrich at cell membranes.

To test our hypothesis, we conducted a smiFISH-based screen in *C. elegans* early embryos for membrane localization of other PIP_2_-binding FERM or PH-like domain-containing transcripts (Nishimura et al., 2015; Tintori et al., 2016). A total of 17 transcripts (nine with FERM domains and eight with PH-like domains) were selected for visualization based on their expression in early embryos and with preference given to homology in other organisms (Table 1; Fig. S4; Fig. S5; Materials and Methods). The set of transcripts selected comprised those that enriched to anterior embryonic cells and to posterior embryonic cells, and those with ubiquitous distribution across the early embryo (Nishimura et al., 2015; Tintori et al., 2016).

**Table 1.**
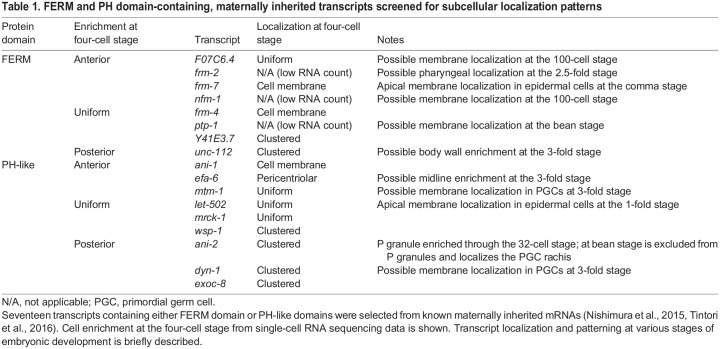
FERM and PH domain-containing, maternally inherited transcripts screened for subcellular localization patterns

**Fig. 5.**
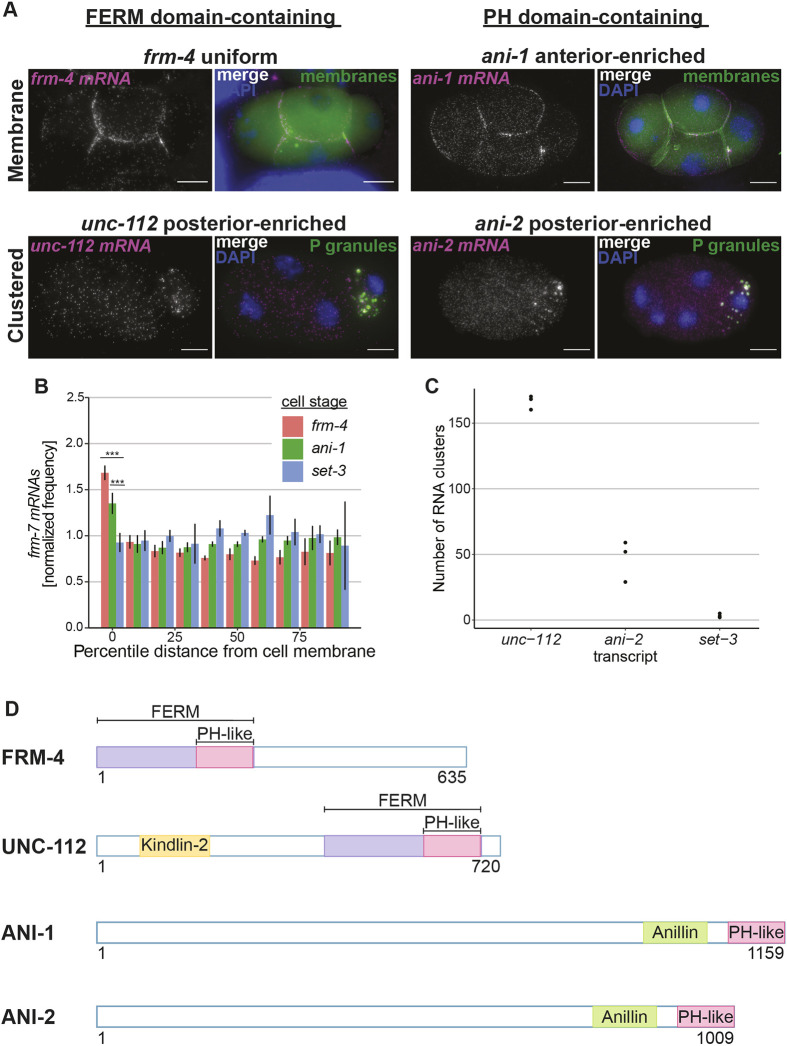
**FERM and PH domain-containing, maternally inherited transcripts display mRNA patterning.** (A) smFISH micrographs of four-cell embryos imaging two FERM domain-containing transcripts, *frm-4* and *unc-112* (magenta, left) and two PH domain-containing transcripts, *ani-1* and *ani-2* (magenta, right). *frm-4* mRNA and *ani-1* mRNA (top) were imaged in a GFP::PH membrane marker transgenic background (membranes, green). *unc-112* mRNA and *ani-12* mRNA (bottom) were imaged in the GLH-1::GFP P granule marker strain (P granules, green). DAPI marks DNA (blue). Scale bars: 10 µm. (B) Quantification of transcripts *frm-4* and *ani-1* compared with the uniform control transcript *set-3* (*n*=3 for each transcript) in four-cell embryos displaying the normalized frequency of *frm-4*, *ani-1* and *set-3* mRNA at increasing, normalized distances from the cell periphery. ***P<0.0005 (Welch two-sample *t*-tests comparing the cell membrane localization for heat shock versus control for the transcript *erm-1* at the given stage). Error bars represent s.d. (C) Total number of clusters detected in four-cell embryos for *unc-112*, *ani-2* and the uniform control *set-3* (*n*=3 for each transcript)*.* (D) Schematics of encoded protein domains for FRM-4, UNC-112, ANI-1 and ANI-2.

Of the 17 screened transcripts**,** three (*frm-4*, *frm-7*, *ani-1*) displayed clear membrane localization in early embryos at the four-cell stage (Fig. 5A,B,D; Fig. S6A,B). Plasma membrane enrichment was 1.8-fold higher for *frm-4* mRNA and 1.5-fold higher for *ani-1* mRNA compared with a *set-3* control mRNA at the four-cell stage (Fig. 5A,B)*.* Although *frm-7* has less obvious localization patterning at the four-cell stage, we quantified localization at the two-cell and four-cell stages and observed a roughly 1.6-fold enrichment at the membrane (Fig. S7A,C). Additionally, at the comma stage *frm-7* mRNA displayed apical enrichment in epidermal cells (Fig. S7B). Seven transcripts had a clustered subcellular patterning in the posterior cell in four-cell-stage embryos (*Y41E3.7*, *unc-112*, *mrck-1*, *wsp-1*, *ani-2*, *dyn-1* and *exoc-8*) (Fig. S6B,C)*.* Of these, *unc-112* and *ani-2* mRNA displayed numerous RNA clusters compared with the uniformly distributed control *set-3* (Fig. 5A,C,D)*.* Five transcripts displayed uniform distribution or other patterns (*F07C6.4*, *efa-6*, *mtm-1*, *mrck-1* and *let-502*) (Fig. S6A,B). Three transcripts (*frm-2*, *nfm-1* and *ptp-1*) yielded RNA abundance too low to determine subcellular enrichment (Table 1; Fig. S6A,B).

Notably, all transcripts previously reported as posterior-cell enriched (Nishimura et al., 2015; Tintori et al., 2016) exhibited clustered patterning in early embryos (Table 1; Fig. S6C). This is consistent with prior visualizations of posterior-enriched transcripts at the four-cell stage that often concentrate within P granules (Parker et al., 2020). Of the transcripts that were uniformly distributed between the anterior and posterior cells [as assayed by previously published RNA sequencing (Nishimura et al., 2015, Tintori et al., 2016)], those with posterior-enrichment below the significance cut-off (*Y43E3.7* and *wsp-1*) also displayed clustered patterning (Table 1; Fig. S6B). Overall, these results support previous findings in the early embryo that AB cell-enriched transcripts tend to localize to membranes, whereas P_1_-enriched transcripts tend to localize to P granules, membraneless organelles housing mRNAs translated at low levels (Lee et al., 2020; Parker et al., 2020; Updike and Strome, 2010). To determine whether clustered transcripts were indeed localizing to P granules or membranes, smFISH was conducted in a P granule or membrane marker strain, respectively. Indeed, even in the case of the homologs *ani-1* and *ani-2*, the anterior-enriched *ani-1* mRNA was membrane localized, whereas the posterior-enriched *ani-2* mRNA localized to P granules (Fig. 5A-C). Importantly, ANI-1 protein is translated in early embryos and concentrates at the plasma membrane whereas early embryo expression of ANI-2 protein is not detected (Maddox et al., 2005). Together, these results suggest that translational dependence of plasma membrane mRNA localization is not limited to *erm-1*.

Our visual screen found that subcellular localization patterns of some transcripts changed over developmental time. At the four-cell stage, *unc-112* mRNA clustered in the P-lineage in P granules (Fig. 5A,C). However, later in embryonic development, at the 3-fold stage, *unc-112* mRNA localized along the body wall where the encoded UNC-112 protein reportedly functions (Fig. S8A) (Rogalski et al., 2000). At the four-cell stage, *F07C6.4* mRNA had uniform distribution (Fig. S6A), but at the 100-cell stage appeared localized to the plasma membranes of a subset of discrete cells (Fig. S8B). Similarly, at the four-cell stage *let-502* had uniform localization (Fig. S4B), but at the 1.5-fold stage it was enriched to the apical membrane of epidermal cells where the encoded LET-502 protein localizes and functions (Fig. S8C) (Piekny et al., 2003). Whereas the *ani-2* transcript clustered in P granules at the four-cell stage (Fig. 5A), at the bean stage and 3-fold stage *ani-2* was excluded from P granules and found at the interface of the primordial germ cells where ANI-2 is found enriched at the rachis bridge of germ cells throughout gonad development (Amini et al., 2014) (Fig. S8D). Overall, ten of the surveyed transcripts without observable membrane localization at the four-cell stage (*F07C6.4*, *frm-2*, *nfm-1*, *ptp-1*, *unc-112*, *efa-6*, *mtm-1*, *let-502*, *ani-2* and *dyn-1*) displayed possible membrane localization at later stages of development, frequently coinciding with where their encoded proteins function (Hunt-Newbury et al., 2007; Josephson et al., 2017; Maddox et al., 2005; Piekny et al., 2003; Rogalski et al., 2000; Thompson et al., 2002; Uchida et al., 2002; Zou et al., 2009). However, the shrinking size of the cells at these stages make it difficult to differentiate membrane enrichment from nuclear exclusion and we therefore label these as ‘possible membrane localization’. Further study will be needed to determine whether these are genuine examples of membrane localization using higher magnification microscopy or biochemical approaches. Overall, our findings suggest that subcellular mRNA patterning is developmentally dynamic.

In total, we identified two FERM domain-encoding transcripts (*frm-4* and *frm-7*) and one PH domain-encoding transcript (*ani-1*), in addition to *erm-1*, that localize to membranes. Because FERM domains contain internal PH domains, we wanted to test whether PH domains were sufficient to direct membrane localization. Using a transgenic GFP::PH strain (Heppert et al., 2016), we used smFISH to assess the localization of *gfp* mRNA (Fig. S9A). This PH domain is from the human phospholipase C delta 1 protein, which localizes to the plasma membrane by binding PIP_2_ (Audhya et al., 2005) and is expressed under the *mex-5* germline promoter. Although the abundance of *gfp::PH* molecules was high (∼10,000 mRNA molecules in a four-cell embryo), nearing the upper limit of our detection capabilities, we observed a roughly 1.3-fold enrichment of *gfp* mRNA molecules at the membrane compared with a uniform control (Fig. S9B,C). Additionally, we observed a gradient pattern concentrically moving away from the plasma membrane, which was not observed in other membrane-localized transcripts. This could be due to the high number of mRNA molecules present in the embryo compared with other transcripts (*erm-1* ∼2400 mRNA molecules/four-cell embryo). Overall, this finding supports a model in which PH domains alone are sufficient to localize their encoding mRNAs.

Our visual screen added to the small but growing list of transcripts that localize to membranes in *C. elegans* (Parker et al., 2020; Tocchini et al., 2021). Thirteen out of the 17 (76%) FERM and PH-like domain-containing transcripts we surveyed exhibited membrane localization or possible membrane localization at some stage during development. However, three of the anterior-enriched transcripts had low RNA abundance at the four-cell stage, complicating our ability to assess their localization. These findings, together with our observation that a PH domain alone is sufficient to direct *gfp* transcript localization, suggest that mRNA membrane localization is a common feature associated with this domain.

## DISCUSSION

**Fig. 6.**
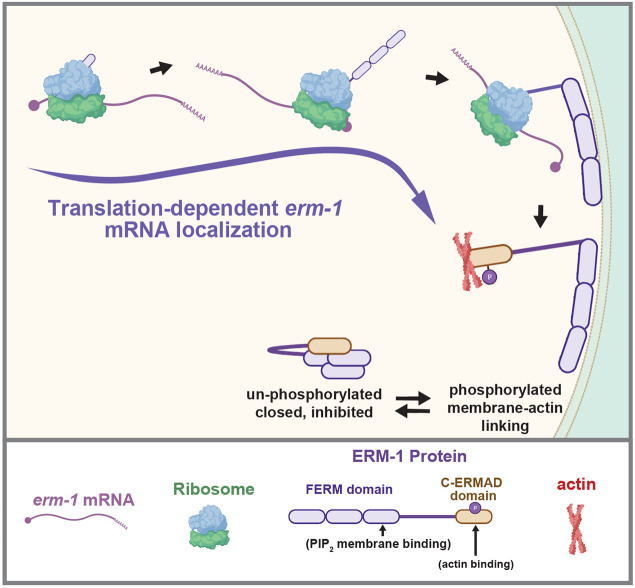
**Model of translation-dependent *erm-1* mRNA localization in the early *C. elegans* embryo.** *erm-1* mRNA is localized in a translation-dependent manner requiring the intact RNC and PIP_2_-binding region of the FERM domain. Local translation of ERM-1 may be important to deposit ERM-1 protein at the proper locale within the cell. Alternatively, local translation of ERM-1 could serve to prevent its autoinhibitory, closed state upon synthesis. Created using BioRender.com.

Here, we report that *C. elegans erm-1* mRNA is localized to the plasma membrane in a translation-dependent manner during early embryonic development (Fig. 6). We showed that in the absence of active translation, an intact RNC is required to maintain *erm-1* mRNA localization. We also demonstrated that, despite mutating 36% of the *erm-1* mRNA sequence, the transcript still localizes properly provided the ERM-1 protein sequence is preserved. This finding further suggests that the localization determinant is specified in the nascent chain of the ERM-1 protein, not as a *cis*-acting element in the mRNA sequence or structure. Furthermore, we narrowed down a domain required for localization and determined that it resided within the FERM domain and depended on the ability of that domain to bind PIP_2_. We identified three additional FERM or PH-like domain-encoding genes (*frm-7*, *frm-4* and *ani-1*) with mRNA localization at the plasma membrane in the early embryo by a smiFISH visual screen and an additional ten genes with mRNA localization patterns later in development that are indicative of possible membrane localization. Our data indicate that subcellular localization is a generalizable feature of transcripts encoding FERM or PH-like domains.

Complementing our findings in the early embryos, studies of later developmental stages identified eight transcripts (*dlg-1*, *ajm-1*, *sma-1*, *vab-10a*, *erm-1*, *pgp-1*, *magu-2* and *let-413*), including *erm-1*, that enrich to regions of the plasma membrane adjacent to apical junctions (Li et al., 2021; Tocchini et al., 2021). In particular, *dlg-1* mRNA localizes through a translation-dependent pathway (Tocchini et al., 2021). *dlg-1* localization requires the translation of N-terminal L27-PDZ domains, C-terminal SH3, Hook and Guk domains to recapitulate fully the localization patterns of the *dlg-1* mRNA. These data, in combination with our findings, suggest that translation-dependent localization could be a prevalent feature of mRNAs that generally encode apical junction components or membrane-associated proteins.

### What is the function of *erm-1* mRNA localization?

An outstanding question is whether *erm-1* mRNA localization is required for ERM-1 function. The ERM-1[4KN] mutation yields *erm-1* mRNA and ERM-1 protein that are mislocalized and results in lethality. However, isolating the function of mRNA localization is problematic because it is very difficult to manipulate mRNA localization while controlling for the pleiotropic effects of perturbing the mRNA. Although this complicates our ability to determine the functional role of mRNA localization, we hypothesize three scenarios. First, it is possible ERM-1 proteins need to be locally translated at the plasma membrane to function properly. Because ERM proteins link the plasma membrane and actin cytoskeleton, local translation could be important for producing ERM-1 linker proteins at the exact sites and at the exact concentrations in which they are needed. Second, we suggest that ERM-1 proteins need to be locally translated at the membrane to prevent negative intramolecular interactions. And third, we suggest that *erm-1* mRNA localization does not serve a role, but is instead a passive result of nascent peptide localization. We will expand on each idea.

It is possible that *erm-1* local translation influences the local abundance of ERM-1 production to direct cell morphology. ERM proteins function in cell movement, membrane trafficking, cell signaling and cell adhesion. They contribute to cancer-associated processes, such as cell metastasis and chemotherapy resistance (Fehon et al., 2010; Kobori et al., 2021; McClatchey, 2003), processes responsive to signaling, polarity or stability cues. Indeed, during embryonic development, the landscape of the plasma membrane is constantly changing and coordination between cell membrane and actin cytoskeletal structures are of paramount importance to cell morphology and cell migration processes. Therefore, local translation of ERM-1 could be sensitive to incoming signals or developmental needs. As an example of this concept, the mRNA encoding PCNT (pericentrin) in human cells and zebrafish embryos localizes co-translationally to dividing centrosomes during early mitosis (Sepulveda et al., 2018). This is hypothesized to supply PCNT protein where it is expeditiously needed for cell division and to mitigate the kinetic challenge of trafficking a large protein.

Our alternative hypothesis for local translation of ERM-1 is that stabilizing the N terminus of ERM-1 at the membrane prevents autoinhibitory interactions. The N-terminal FERM domain and C-terminal C-ERMAD domains can intramolecularly bind to promote an inhibited state that relocalizes to the cytoplasm. This auto-inhibition is prevented through phosphorylation of C-ERMAD (Li et al., 2007; Pearson et al., 2000; Roch et al., 2010). Perhaps local translation of ERM-1 at the membrane assists in counteracting autoinhibition until C-ERMAD phosphorylation occurs. By this logic, ERM proteins could be translated in an ‘ON’ state, ready to perform their function.

Finally, it is possible that localization of *erm-1* mRNA is not functional, but is instead the result of the FERM domain of ERM-1 localizing concomitantly with translation. In this scenario, the mRNA is passively carried to the plasma membrane. If we could tether mRNAs to different locations, perhaps we could test this scenario.

### Which pathways localize ERM-1 RNCs to the plasma membrane in the early embryo?

The pathway that directs (transmembrane protein- and secretory protein-encoding) mRNAs to the ER is the most well-characterized translation-dependent mRNA trafficking pathway and requires the presence of a signal peptide. As ERM-1 lacks a discernable ER-directing signal peptide and fails to pull down ER associated-components in immunoprecipitation assays, we surmise that the pathway that directs translating *erm-1* to the plasma membrane is likely distinct from the ER secretory pathway (Ast et al., 2013; Cohen et al., 2020; Keenan et al., 2001). Alternatively, if *erm-1* is localized in an ER-dependent pathway it would likely be noncanonical.

It is theoretically possible that localization of ERM-1 RNCs occurs through either passive diffusion or active trafficking. We argue that passive diffusion is unlikely as a recent study that performed live imaging of *erm-1* mRNA using the PP7-PCP system found that *erm-1* mRNA movements were rapid, directional and dynein motor (DHC-1) dependent, suggesting that *erm-1* localization requires active cytoskeletal trafficking (Li et al., 2021). Future studies will aim to identify the molecular machinery involved in the transport of *erm-1* mRNA, such as potential protein effectors that bind its nascent chain. However, it is possible that the mechanism of *erm-1* mRNA localization is dynamic during development and that active localization is required during early development given the volume of the cells and speed of division, whereas diffusion and anchoring could occur at later stages when cells are smaller and polarized.

It will be interesting to determine whether *erm-1* translation is paused or active during the trafficking process and whether multiple rounds of translation occur at the membrane. A pause in translation or multiple rounds of translation could explain why *ani-1* mRNA localizes to membranes although the encoded PH domain is C-terminal. Future studies that perform nascent chain tracking to perform live imaging of where nascent ERM-1 peptides are produced will be instrumental in determining how localization and translational initiation are linked (Morisaki et al., 2016).

### What is the status of the cytosolic population of *erm-1* mRNA?

We observed that roughly 40% of *erm-1* mRNA localize to the plasma membrane versus 60% to the cytosol at the four-cell stage of development. Therefore, there are both cytoplasmic and plasma membrane-localized populations of *erm-1* mRNAs. We speculate that cytoplasmic *erm-1* mRNA transcripts may not be undergoing active translation or are at the beginning of translation, prior to the production of the FERM domain. They are likely not in a ribosome-free state because we do not observe them in a clustered pattern in either the somatic or germ cells, where they would be stored or degraded in P granules, P bodies or stress granules. This is another area in which nascent chain tracking live imaging technology may yield greater insight in future studies. The recently developed technique ‘fluorescence assay to detect ribosome interactions with mRNA’ (FLARIM) would also be useful for addressing this question (Richer et al., 2021 preprint).

### How do *erm-1* mRNA and ERM-1 protein localize in polarized cells?

ERM-1 proteins localize to plasma membranes generally. However, during mid-embryogenesis, ERM-1 concentrates at the apical side of polarized intestinal cells to coat the intestinal lumen. This apical enrichment occurs first with ERM-1 proteins localizing to the plasma membrane in a FERM-dependent step and subsequently with re-localization to the apical face in a C-ERMAD-dependent step (Ramalho et al., 2020) (Fig. 4D). During these phases, we found that *erm-1* mRNA localized to the plasma membrane along with ERM-1 protein in the first localization step but failed to concentrate at the apical membrane at the second, relocalization step. This suggests that ERM-1 protein re-localization to the apical surface likely occurs post-translationally.

Our screen of FERM and PH-like domain-containing genes yielded multiple transcripts with mRNA localization at the plasma membrane, suggesting that this property is conserved across species. The *ani-1* and *ani-2* ortholog in *Drosophila*, *anillin*, concentrates at the pseudo-cleavage furrow of *Drosophila* embryos dependent on translation of both its PH- and actin-binding domains (Hirashima et al., 2018). Indeed, the expanded use of mRNA imaging and subcellularly enriched RNA-sequencing technologies has led to a greater appreciation that localization of mRNA is a widespread feature of cell biology, not only to the plasma membrane, but to a wide diversity of membranes and other subcellular structures (Chouaib et al., 2020; Safieddine et al., 2021). These findings suggest that many proteins may benefit from local translation at their destination site.

## MATERIALS AND METHODS

### Worm husbandry

*C. elegans* strains were cultured according to standard methods (Brenner, 1974). Worm strains were maintained and grown at 20°C on nematode growth medium (NGM: 3 g/l NaCl, 17 g/l agar, 2.5 g/l peptone, 5 mg/l cholesterol, 1 mM MgSO_4_, 2.7 g/l KH_2_PO_4_, 0.89 g/l K_2_HPO_4_). Strains used in this study are listed in Table S1.

### Heat-shock experiments

Heat-shock experiments were performed by transferring harvested embryos to pre-warmed M9 liquid media and incubating at 30°C for 25 min. After heat shock, worms were immediately fixed for smFISH.

### RNAi feeding for smFISH microscopy

RNAi feeding constructs were obtained from the Ahringer library (Fraser et al., 2000). Bacteria containing inducible dsRNA vectors were grown at 37°C in LB+ampicillin (100 µg/ml) media for 16 h, spun down and resuspended at 10× original concentration with M9, plated on NGM+carbenicillin (100 µg/ml)+IPTG (1 mM) plates, and grown at room temperature overnight, or until plates were dry. Embryos were harvested for synchronization from mixed staged worms. Harvested embryos were incubated in M9 for 24 h at room temperature while nutating until all arrived at L1 developmental stage. L1 worms were deposited on RNAi feeding plates and grown at 20°C for 48 h. Embryos were harvested from gravid adults and smFISH was conducted. For each gene targeted by RNAi, we performed at least three independent replicates. L4440 RNAi empty vector was used as a negative control and *pop-1* RNAi used as an embryonic lethal positive control. For experiments performing *ifg-1* RNAi, synchronized L1 worms were grown to L2 on OP50 plates before being washed in M9 and transferred to RNAi plates for 48 h. *ifg-1* RNAi resulted in embryonic lethality. *Escherichia coli* strains used in this study can be found in Table S2.

### Permeabilization and drug delivery

For small molecule inhibitor treatment, *perm-1* RNAi was performed to permeabilize the eggshell as described by Carvalho et al. (2011). Briefly, synchronized L1 worms were fed on RNAi for 48 h and embryos were hand-dissected from the treated mothers. Permeabilization by *perm-1* RNAi was tested by submerging embryos in water to induce bursting by the increased internal osmotic pressure. Additionally, RNAi efficacy was confirmed using a *pop-1* RNAi positive control. Small molecule inhibitor treatment was based on the protocol described by Lee et al. (2020). To harvest embryos, young adult worms were washed off plates in 5 ml S-buffer (129 ml 0.05 M K_2_HPO_4_, 871 ml 0.05 M KH_2_PO_4_, 5.85 g NaCl, 300±5 mOsm) and allowed to settle to the bottom of a 15 ml conical flask (no longer than 5 min). S-buffer was removed, and worms were resuspended in 100 µl S-buffer alone (negative control) or drug diluted in 100 µl S-buffer (500 µg/ml cycloheximide or 100 µg/ml puromycin final concentrations). The 100 µl drug solution and young adult worms were transferred to a concavity slide, hand-dissected in a concavity slide, transferred to a 1.5 ml Eppendorf tube and incubated for 20 min. Both puromycin and cycloheximide treatments induced cell cycle arrest as observed by Lee et al. (2020). After incubation with drug solution, 1 ml of −20°C methanol was added to fix the embryos. Embryos were freeze-cracked in liquid nitrogen for 1 min then incubated overnight in methanol at −20°C to continue the fixation. smFISH was performed as described. Owing to the fragility of *perm-1* depleted embryos, all spins required in their smFISH preparation were performed at 250 ***g*** instead of 2000 ***g***.

### Synonymous *erm-1* strain generation

The synonymous *erm-1* strain (wLPW008) and the associated control strain (wLPW007) were generated by InVivo Biosystems. The control strain (wLPW007) was designed using a modified *erm-1* cDNA sequence that generated both the a and b isoforms driven off the *erm-1* promoter and incorporated artificial introns to prevent germline silencing. The synonymous strain (wLPW008) was generated from the control sequence using a proprietary algorithm (InVivo Biosystems) and alternative, worm-optimized codons were introduced to ensure comparable expression. Both constructs were inserted as a single copy at the chromosome IV MosSCI locus cxTi10882.

### smFISH

smFISH was performed based on the TurboFish protocol (Femino et al., 1998; Nishimura et al., 2015; Parker et al., 2021; Raj and Tyagi, 2010; Raj et al., 2008; Shaffer et al., 2013). Updates specific to *C. elegans* were made using new Biosearch reagents (outlined by Parker et al., 2021). Using the Stellaris RNA FISH Probe Designer, custom FISH probes were designed for transcripts of interest (Parker et al., 2020, 2021) (Biosearch Technologies), available online at www.biosearchtech.com/stellarisdesigner (version 4.2). Embryos were hybridized with Stellaris RNA FISH Probe sets labeled with CalFluor 610 or Quasar 670 (Biosearch Technologies), following the manufacturer's instructions (see www.biosearchtech.com/stellarisprotocols). Briefly, adult worms were bleached for embryos, suspended in 1 ml −20°C methanol, freeze-cracked in liquid nitrogen for 1 min, and fixed overnight in methanol at −20°C for 1-24 h. Alternatively, fixation was carried out for 4 min followed by the 1 min freeze crack, switching to −20°C acetone incubation for an additional 5 min. ERM-1::GFP worms were freeze-cracked in 1 ml acetone followed by a 35 min incubation at −20°C. After fixation, embryos incubated in Stellaris Wash Buffer A for 5 min at room temperature (Biosearch Technologies, SMF-WA1-60) before hybridization in 100 µl Stellaris Hybridization buffer (Biosearch Technologies, SMF-HB1-10) containing 50 pmols of each primer set (up to two channels per experiment) and 10% formamide for 16-48 h at 37°C with mixing at 400 rpm in a thermomixer (Eppendorf ThermoMixer F1.5). Embryos were washed for 30 min in Stellaris Wash Buffer A, followed by a second wash of Stellaris Wash Buffer A containing 5 µg/ml DAPI, then 5 min in Wash Buffer B followed by a second 5 min wash (Biosearch Technologies, SMF-WB1-20) before incubation in N-propyl gallate antifade (10 ml 100% glycerol, 100 mg N-propyl gallate, 400 µl 1 M Tris pH 8.0, 9.6 ml DEPC-treated H_2_O) prior to slide preparation. All embryos were centrifuged in spin steps at 2000 ***g*** unless otherwise noted. Embryos were mounted using equal volumes hybridized embryos resuspended in N-propyl gallate antifade and Vectashield antifade (Vector Laboratories, H-1000). smFISH image stacks were acquired as described by Parker et al. (2020) on a Photometrics Cool Snap HQ2 camera using a DeltaVision Elite inverted microscope (GE Healthcare), with an Olympus PLAN APO 60× (1.42 NA, PLAPON60XOSC2) objective, an Insight SSI 7-Color Solid State Light Engine, and softWoRx software (Applied Precision) using 0.2 m *z*-stacks. DeltaVision (softWoRx) deconvolution software was applied for representative images. Images were further processed using Fiji (Schindelin et al., 2012). For each condition, a minimum of five embryos per four-cell stage were analyzed, but often many more across multiple cell stages were imaged. All smFISH and smiFISH probes can be found in Table S3. All raw microscopy images have been deposited in Mountain Scholar, a digital, open-access data repository associated with Colorado State University Libraries.

### smiFISH

smiFISH was performed as described by Parker et al. (2021). Briefly, custom primary DNA oligonucleotides were designed as described (Tsanov et al., 2016) complementary to the 17 FERM and PH-like domain-containing transcripts screened and ordered from IDT (https://www.idtdna.com/pages/products/custom-dna-rna/dna-oligos) (Table S3). Secondary FLAPX probes were ordered with dual 5′ and 3′ fluorophore labeling, Cal Fluor 610 or Quasar 670, from Stellaris LGC (Biosearch Technologies, BNS-5082 and FC-1065, respectively). Secondary, fluorophore-labeled probes were annealed to primary probes fresh for every experiment in a thermocycler at 85°C for 3 min, 65°C for 3 min and 25°C for 5 min.

### Quantification of plasma membrane RNA localization

Quantification of transcript localization with reference to the cell membrane was performed as previously described (Parker et al., 2020) using the web application ImJoy (Ouyang et al., 2019). Briefly, RNAs were first detected from raw images using the MATLAB code FISH-quant (Mueller et al., 2013). Individual cell outlines were then manually annotated in Fiji for each *z*-stack in the micrograph, excluding the uppermost and lowermost stacks where cells were flattened against the slide or coverslip or there was out-of-focus light. The distance of each RNA was then measured from the nearest annotated membrane and binned in 10% distance increments away from nearest membrane to account for any change in size between embryos. The total numbers of RNAs per bin were then normalized by the volume of the concentric areas they occupied. After this normalization, values greater than 1 indicated that for this distance more RNAs were found compared with a randomly distributed sample. Source data for figure generation and quantification are available from the Dryad Digital Repository (Winkenbach et al., 2022): dryad.9cnp5hqnp. The source code for quantification described by Parker et al., 2020 can be found at https://github.com/muellerflorian/parker-rna-loc-elegans.

### Quantification of total mRNA

Detection of RNA molecules was performed in the 3D image stacks with FISH-quant (Mueller et al., 2013). Post-processing to calculate the different location metrics was performed as described above with custom written MATLAB and Python codes. The Python code was implemented in user-friendly plugins for the image processing platform ImJoy (Ouyang et al., 2019). Source data for figure generation and quantification are available from the Dryad Digital Repository (Winkenbach et al., 2022): dryad.9cnp5hqnp. The source code for quantification described by Parker et al., 2020 can be found at https://github.com/muellerflorian/parker-rna-loc-elegans.

To quantify the number of individual mRNAs in the ERM-1[4KN] strain, a custom MATLAB script was implemented. FISH-quant detection settings were used to identify candidate mRNA clusters from smFISH micrographs using a Gaussian Mixture Model (GMM). The GMM differentiates independent, single mRNAs from groups of clustered mRNAs by probabilistically fitting a predicted RNA of average intensity and size over each FISH-quant-detected RNA.

### Domain search

A smiFISH-based screen was utilized to identify whether transcripts with protein domains similar to *erm-1* also displayed membrane localization. WormBase ParaSite was utilized to generate a list of proteins with domain IDs matching those annotated for ERM-1. Proteins with domain IDs matching ERM-1 were subset for genes present in two-cell-stage embryos (Nishimura et al., 2015). This resulted in a list of 149 maternally inherited genes encoding either a FERM or PH-like domain. (Table S4). Based on single-cell sequencing at the two-cell stage (Nishimura et al., 2015) up to the 16-cell stage (Tintori et al., 2016), these transcripts have known patterns of anterior, posterior or uniform distribution between the cells. Seventeen transcripts were selected from the total of 149 candidate genes based on distribution at the four-cell stage, RNA count and available expression data on WormBase.

Candidate genes were further subset using the ‘Interactive visualizer of differential gene expression in the early *C. elegans* embryo’ (http://tintori.bio.unc.edu/; Tintori et al., 2016). Candidate genes were manually curated based on the following criteria: (1) persistence of enrichment in the four-cell-stage embryo, (2) high transcript abundance in the four-cell-stage embryo, (3) homology to genes encoding transcripts with known localization in other biological systems, and (4) existing protein expression data available on WormBase (https://wormbase.org/). Manual curation resulted in 17 candidate genes that were simultaneously maternally inherited and contained FERM or PH-like domains to screen for membrane localization (Table 1).

## Supplementary Material

10.1242/develop.200930_sup1 Supplementary information
