# Supplementary material for: The ERM-1 membrane-binding domain directs erm-1 mRNA localization to the plasma membrane in the C. elegans embryo
Source: Development. 2022 Nov 21;149(22):dev200930. doi: 10.1242/dev.200930 (PMC9793419; doi:10.1242/dev.200930)
Supplement: Supplementary information [file develop-149-200930-s1.pdf]

Figure S1

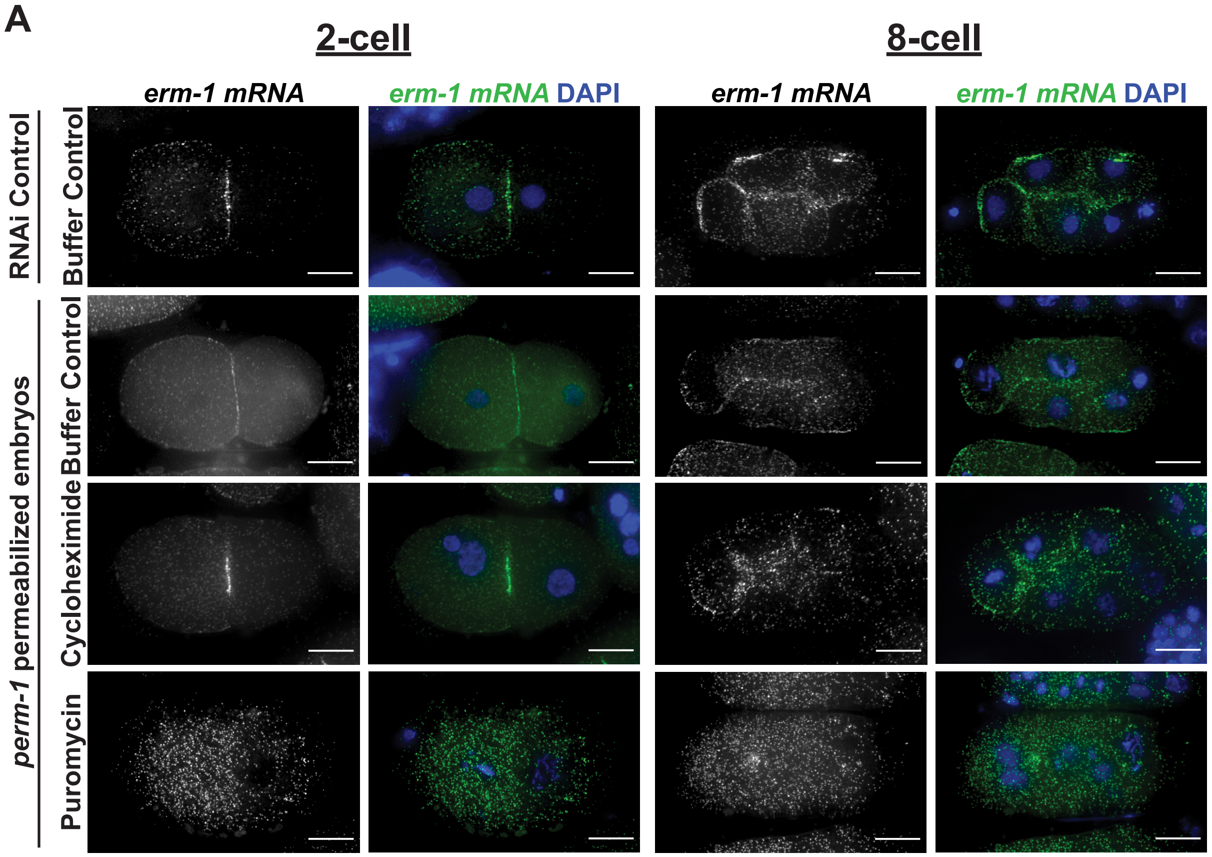

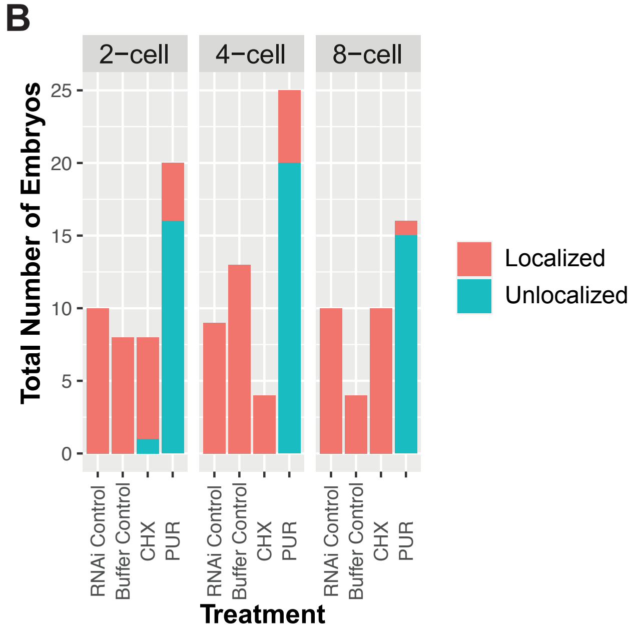

Development • Supplementary information

Fig. S1. The ERM-1 nascent peptide is required for *erm-1* mRNA cell membrane localization. (A) Fluorescent micrographs of 2-cell and 8-cell *C. elegans* embryos are shown in which embryos were permeabilized by *perm-1* RNAi and subsequently treated with small molecule translation inhibitors, in comparison to RNAi and drug treatment controls. *erm-1* mRNA (green) was imaged by smFISH, under control, cycloheximide (500 µg/mL, 20min), or puromycin (500 µg/mL, 20min) treatment conditions. Scale bars 10 µm. **(B)** Bar plot indicating the number of embryos displaying *erm-1* mRNA enriched at cell membranes (localized) or homogenously distributed through the cell (unlocalized) for 2-cell, 4-cell, and 8-cell embryos subjected to the indicated treatments.

Figure S2

A

Figure 3 worm strains		
erm-1::gfp (endogenous)	*erm-1p::erm-1::gfp::erm-1 3'UTR* I	*gfp* knock-in at endogenous erm-1 locus on Chr I (Ramalho et al., 2021)
erm-1::gfp (transgene)	*erm-1p::erm-1::gfp::erm-1 3'UTR* IV	single copy insertion at Chr IV MosSCI locus (this study)
erm-1 synon::gfp (transgene)	*erm-1p::erm-1 synon::gfp::erm-1 3'UTR* IV	single copy insertion at Chr IV MosSCI locus (this study)

B

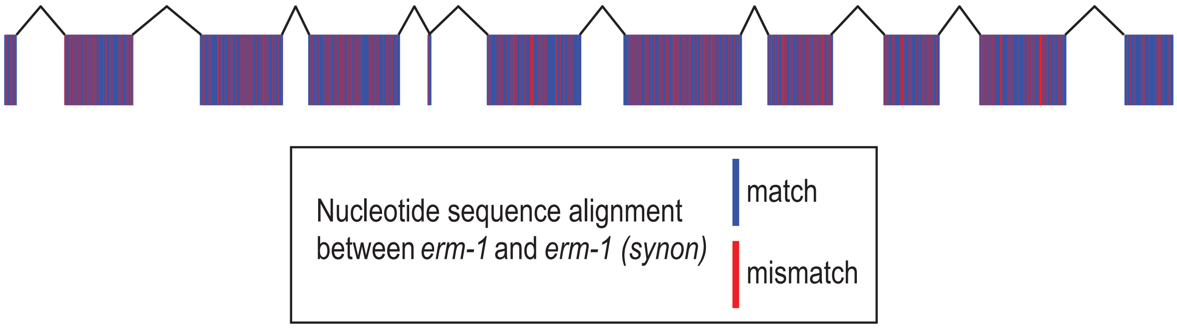

C

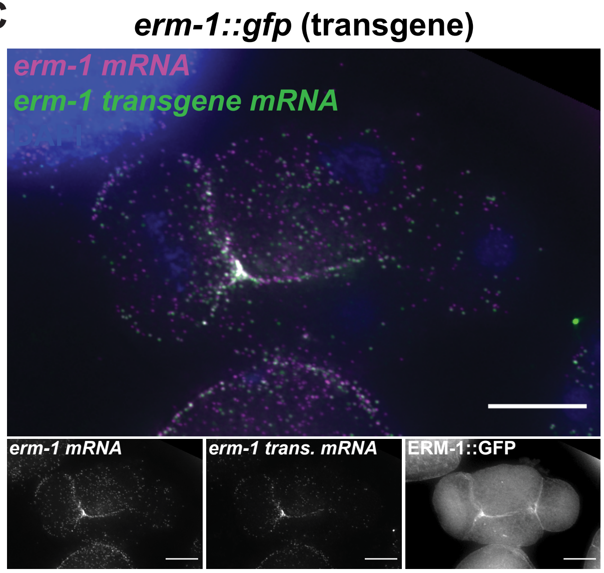

D

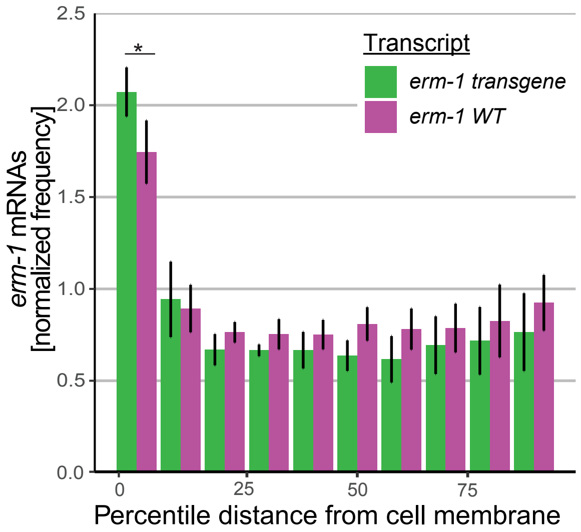

Development • Supplementary information

Fig. S2. *erm-1* mRNA localizes at the cell membrane when expressed from a transgenic MosSCI locus. (A) Description of the three worm strains used in the synonymous *erm-1* assays. **(B)** nucleotide sequence alignment between *erm-1* and the synonymous *erm-1(erm-1 (synon))* showing nucleotide matches in blue and mismatches in red. **(C)** smFISH micrographs of 4-cell staged *C. elegans* embryos imaging *erm-1::gfp* transgene probed by *erm-1* (*erm-1 mRNA,* magenta) and the transgenic *erm-1::gfp* mRNA probed by *nemametrix gfp* (*erm-1 transgene mRNA,* green). DNA (DAPI, blue) and membranes marked by ERM-1::GFP expressed at the MosSCI locus are also shown. **(D)** Quantification of endogenous and MosSCI-expressed transgenic *erm-1* mRNA (n = 6) indicating the volume normalized frequency of mRNAs within binned, normalized distances from the cell membrane. Welch Two Sample t-test p-value = 0.006 at the cell membrane. Scale bars 10 µm. *P* value legend: NS>0.05; 0.05>*>0.005; 0.005>**>0.0005; 0.0005>***>0.00005; 0.00005>****

Figure S3

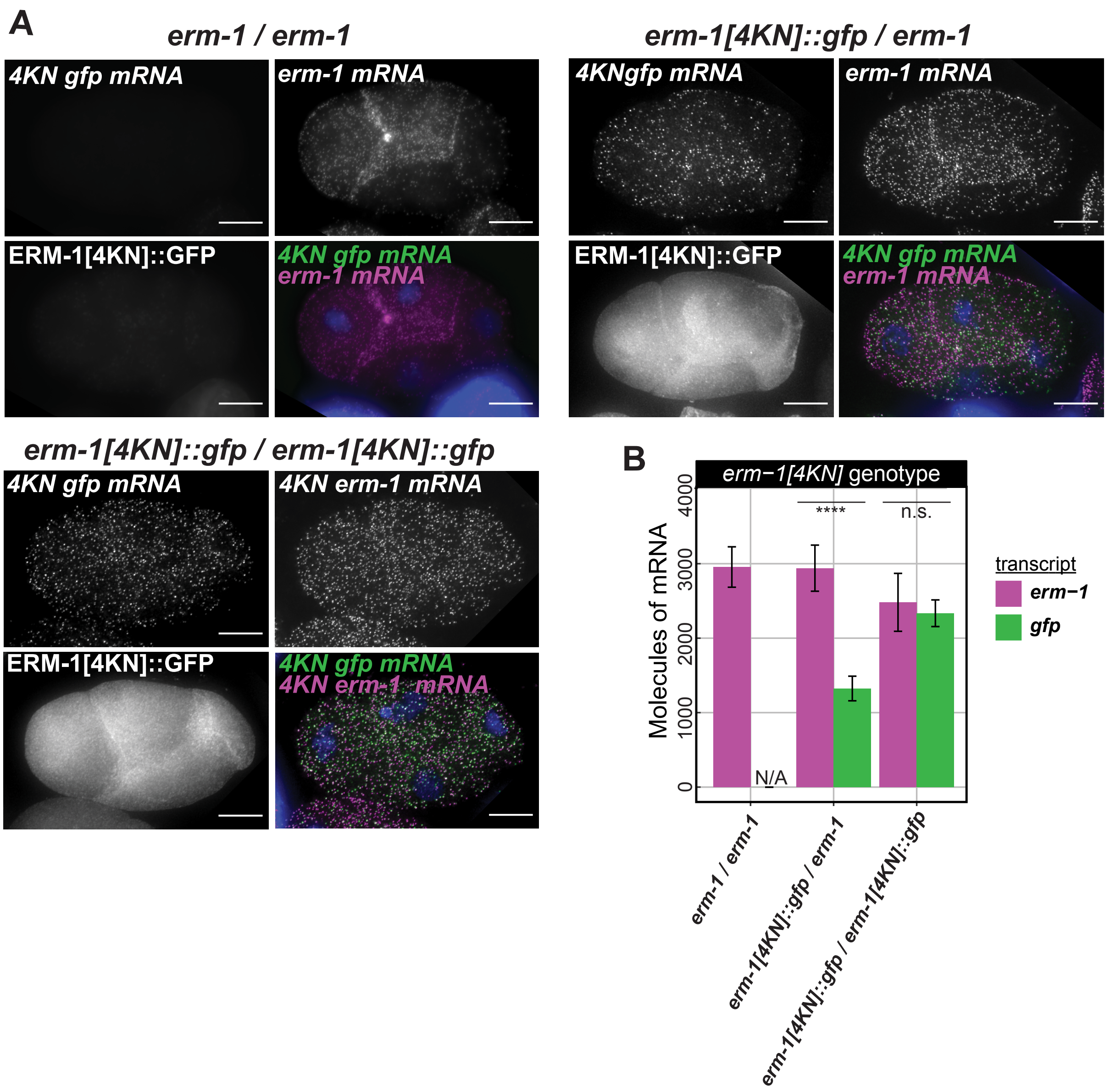

Development • Supplementary information

Fig. S3. ERM-1[4KN]::GFP RNA abundance corresponds to genomic copy-number. (A) 4-cell embryos of the ERM-1[4KN]::GFP mutant probing for *erm-1* mRNA (magenta) and *4KN gfp* (green) are shown in *erm-1/erm-1* homozygotes (*n* = *9*), *erm-1[4KN]::gfp/erm-1* heterozygotes (*n* = *9*), or *erm-1[4KN]::gfp/erm-1[4KN]::gfp* homozygous mutants (*n* = *9*). Scale bars 10 µm. **(B)** Total number of *erm-1* and *gfp* mRNA molecules *P*-values derived from Welch Two Sample *t*-tests comparing the number of *gfp* and *erm-1* mRNA molecules detected in the same strains. *P* value legend: NS>0.05; 0.05>*>0.005; 0.005>**>0.0005; 0.0005>***>0.00005; 0.00005>****

Figure S4

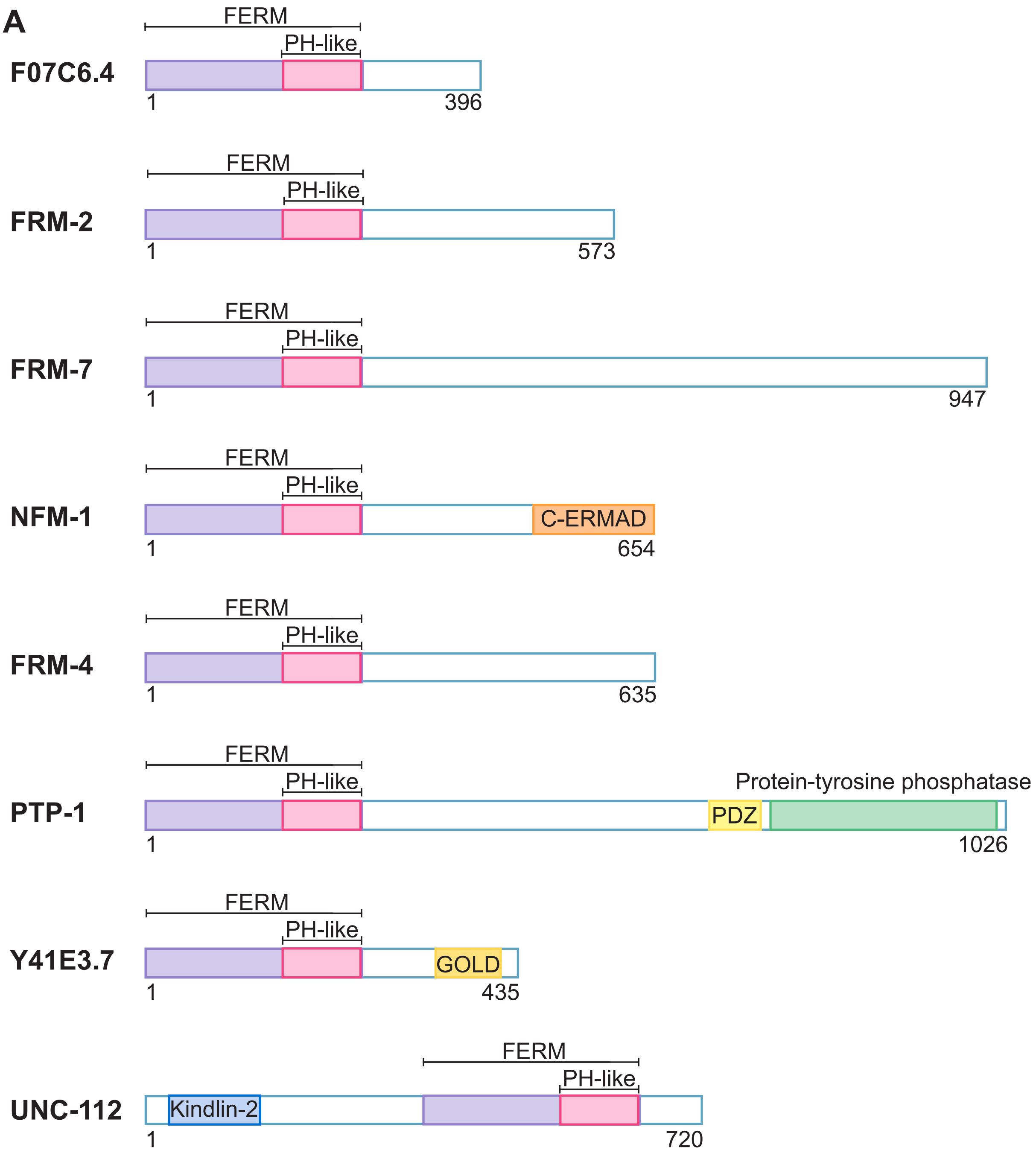

Fig. S4. Depictions of the encoded protein domains for the surveyed FERM domain-containing transcripts.

Development • Supplementary information

Figure S5

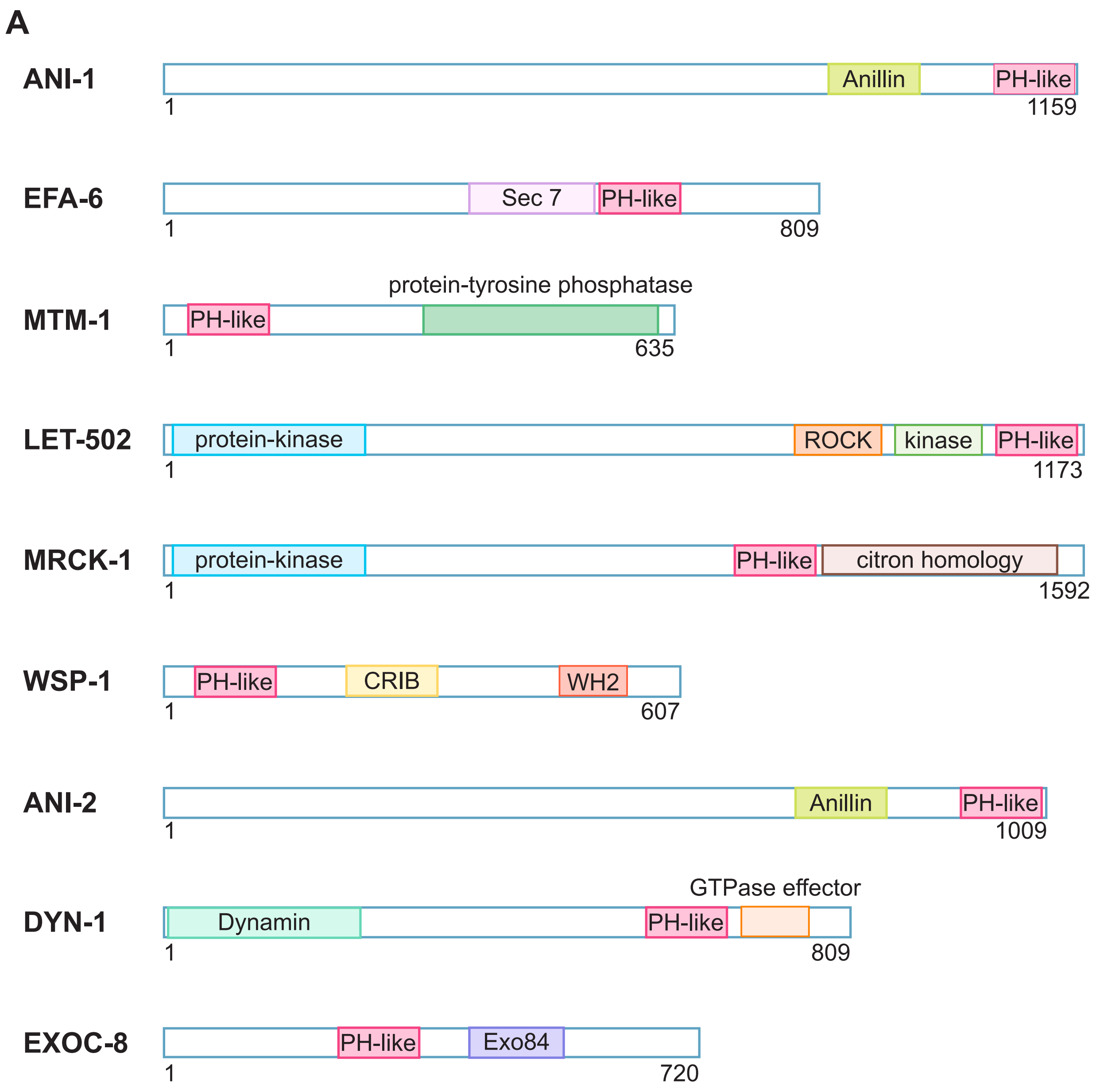

Fig. S5. Depictions of the encoded protein domains for the surveyed PH-like domain-containing transcripts.

Development • Supplementary information

Figure S6

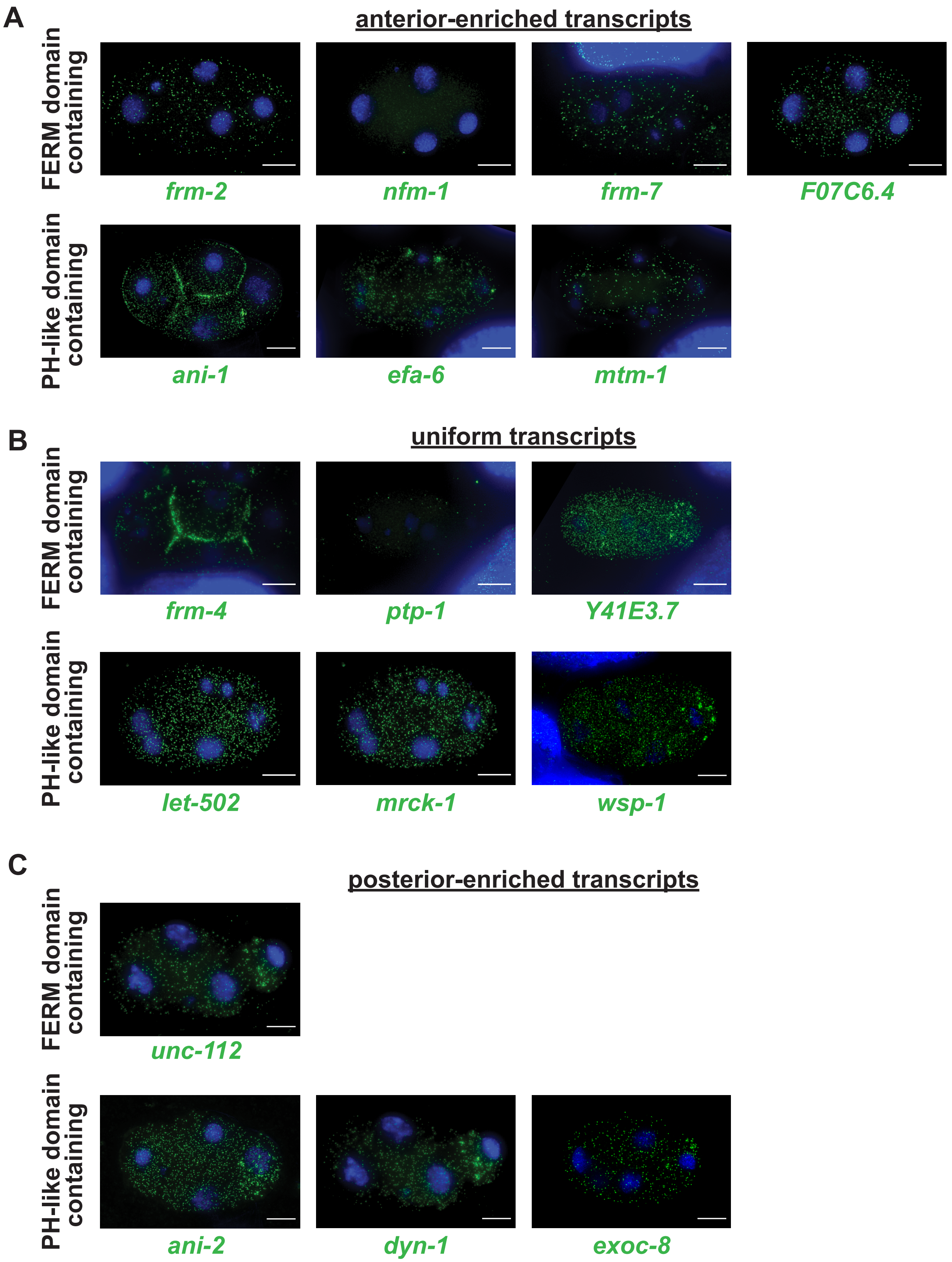
A
anterior-enriched transcripts
FERM domain containing
frm-2
nfm-1
frm-7
F07C6.4
PH-like domain containing
ani-1
efa-6
mtm-1
B
uniform transcripts
FERM domain containing
frm-4
ptp-1
Y41E3.7
PH-like domain containing
let-502
mrck-1
wsp-1
C
posterior-enriched transcripts
FERM domain containing
unc-112
PH-like domain containing
ani-2
dyn-1
exoc-8

Fig. S6. Subcellular localization patterns of surveyed FERM and PH domain-containing transcripts. (A) Seven anterior-enriched transcripts *F07C6.4, frm-2, frm-7, nfm-1, ani-1, efa-6,* and *mtm-1,* **(B)** six uniformly distributed transcripts *frm-1, ptp-2, Y41E3.7, let-502, mrck-1,* and *wsp-1,* and **(C)** four poster-enriched transcripts *unc-112, ani-2, dyn-1,* and *exoc-8* are shown*.*

Development • Supplementary information

Figure S7

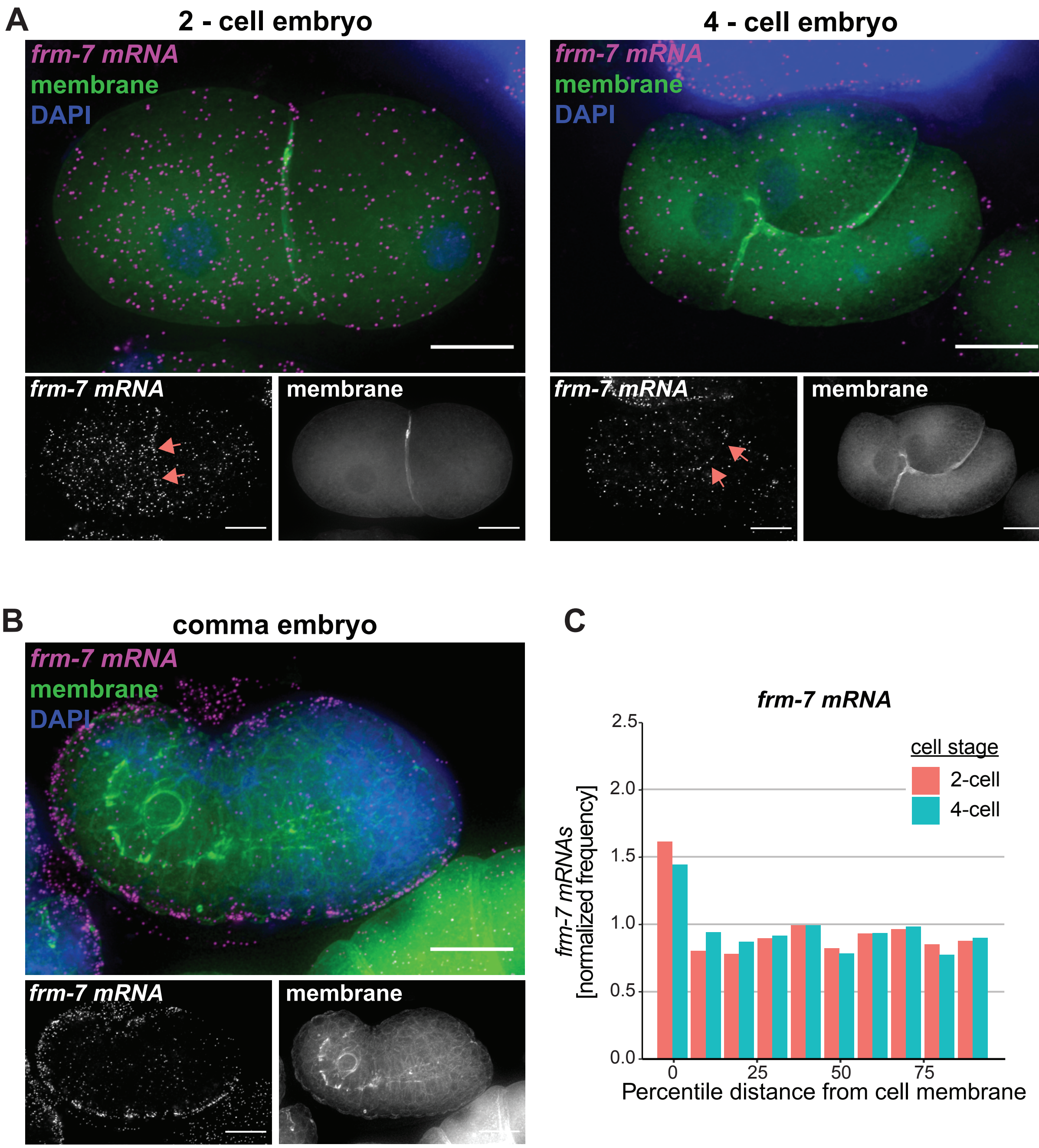
A
2 - cell embryo
4 - cell embryo
frm-7 mRNA
membrane
DAPI
frm-7 mRNA
membrane
B
comma embryo
frm-7 mRNA
membrane
DAPI
frm-7 mRNA
membrane
C
frm-7 mRNA
cell stage
2-cell
4-cell
frm-7 mRNAs [normalized frequency]
2.5
2.0
1.5
1.0
0.5
0.0
0
25
50
75
Percentile distance from cell membrane

Fig. S7. Subcellular localization patterns of *frm-7* in early and mid-stage embryos. **(A)** smFISH micrographs of a 2-cell and 4-cell embryo imaging the anterior-enriched, FERM domain encoding transcript *frm-7* mRNA (magenta) in a GFP::PH membrane marker transgenic background (GFP::PH, green)**.** **(B)** smFISH micrograph of a comma stage *C. elegans* embryo imaging the FERM domain encoding transcript *frm-7* (magenta) in ERM-1:GFP background (green) with DNA (DAPI, blue). **(C)** Quantification of *frm-7* mRNA in a 2-cell embryo (*n=1*) and 4-cell embryo (*n=1*) indicating the normalized frequency frm-7 mRNA at increasing, normalized distances from the cell periphery.

Development • Supplementary information

Figure S8

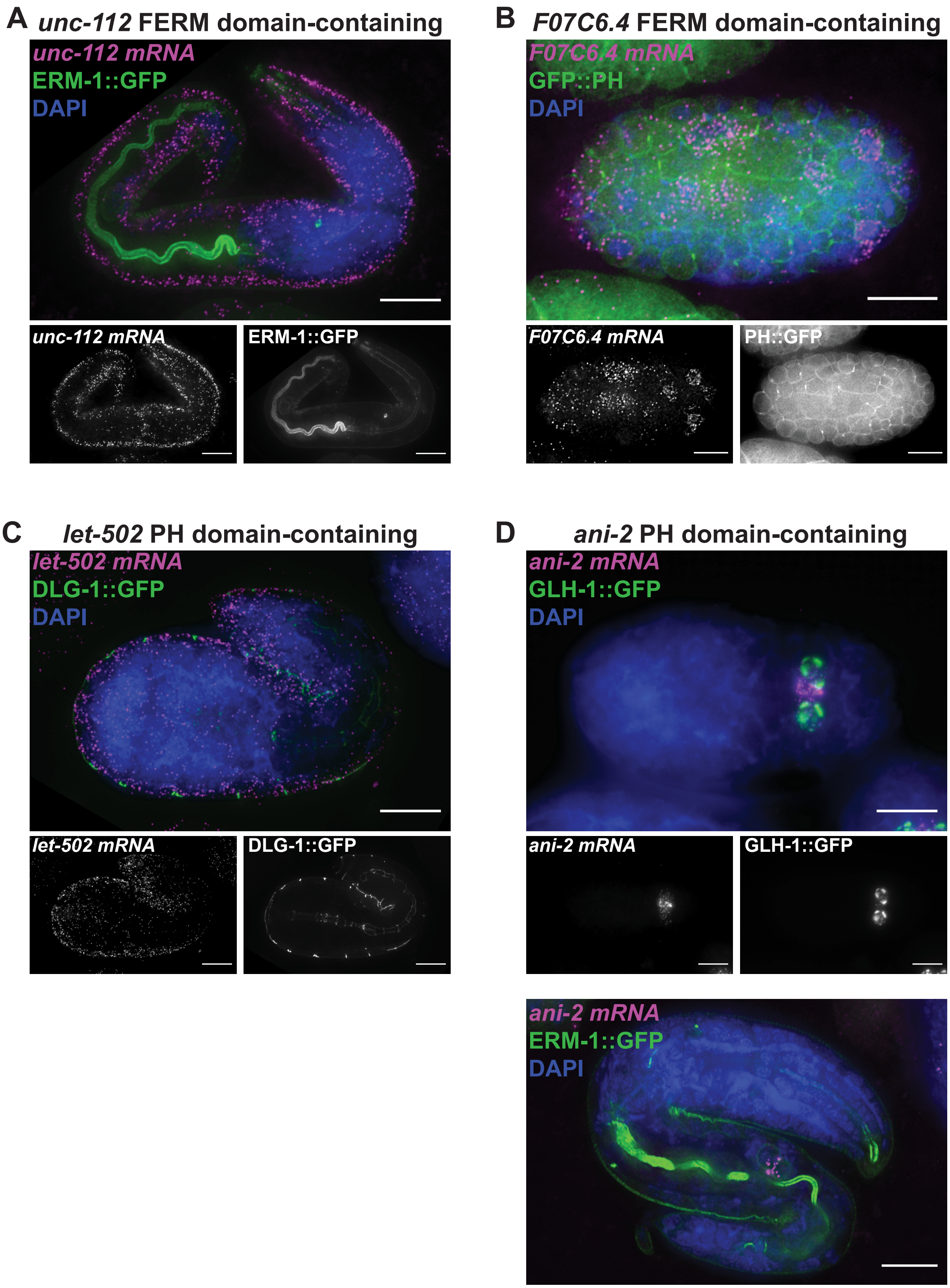
A *unc-112* FERM domain-containing
unc-112 mRNA
ERM-1::GFP
DAPI
unc-112 mRNA
ERM-1::GFP
B *F07C6.4* FERM domain-containing
F07C6.4 mRNA
GFP::PH
DAPI
F07C6.4 mRNA
PH::GFP
C *let-502* PH domain-containing
let-502 mRNA
DLG-1::GFP
DAPI
let-502 mRNA
DLG-1::GFP
D *ani-2* PH domain-containing
ani-2 mRNA
GLH-1::GFP
DAPI
ani-2 mRNA
GLH-1::GFP
ani-2 mRNA
ERM-1::GFP
DAPI

Fig. S8. Subcellular localization patterns of surveyed FERM and PH domain-containing transcripts at mixed later stages. (A) smFISH micrograph of 3-fold *C. elegans* embryo imaging the posterior-enriched, FERM domain encoding transcript *unc-112* (magenta) in ERM-1:GFP background (green) with DNA (DAPI, blue). **(B)** smFISH micrograph of 100-cell *C. elegans* embryo imaging the anterior-enriched, FERM domain encoding transcript *F07C6.4* (magenta) in GFP::PH membrane marker background (green) with DNA (DAPI, blue). **(C)** smFISH micrograph of 1.5-fold *C. elegans* embryo imaging the uniformly-distributed, PH-like domain encoding transcript *let-502* (magenta) in DLG-1:GFP background (green) with DNA (DAPI, blue). **(D)** smFISH micrograph of a bean (top) and 3-fold (bottom) *C. elegans* embryo imaging the posterior-enriched, PH-like domain encoding transcript *ani-2* (magenta) in GLH-1::GFP P granule marker background (green, top) and ERM-1:GFP background (green, bottom) with DNA (DAPI, blue).

Figure S9

A

sufficiency experiment worm strain		
gfp:: PLc PH (SCI transgene)	*mex-5p::gfp::PLc PH::tbb-2 3'UTR*	GFP fused to PH domain from phospholipase C-(delta)1 single copy insertion (Heppert et al., 2016)

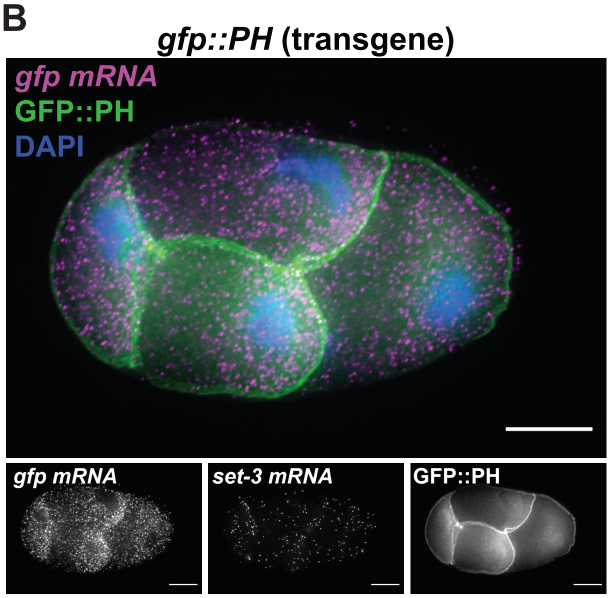

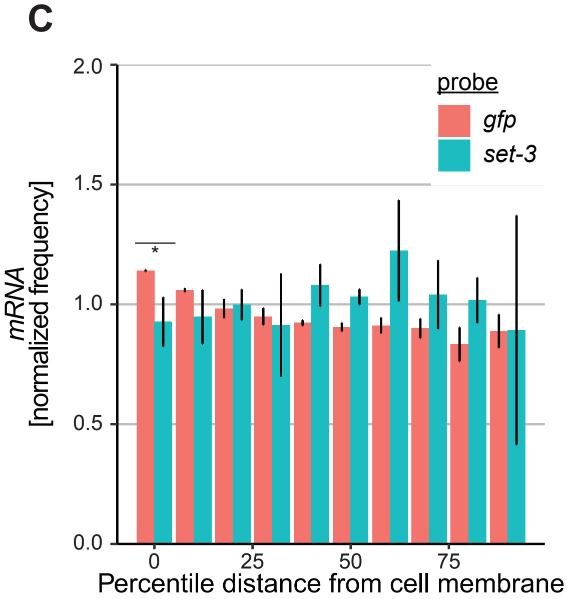

Fig. S9. Sufficiency of the phospholipase C-delta-1 PH domain to direct *gfp* localization to plasma membranes at the 4-cell stage. (A) description of the GFP::PH reporter construct. (B) smFISH micrographs of 4-cell embryos imaging *gfp::PH* by probing *gfp*, (magenta) in a GFP::PH membrane marker transgenic background (GFP::PH, green). **(C)** Quantification of transcripts *frm-4* and *ani-1* compared to uniform control transcript *set-3* (for each transcript *n=3*) in 4-cell embryos displaying the normalized frequency of *frm-4, ani-1,* and *set-3* mRNA at increasing, normalized distances from the cell periphery. Significance indicates *P*-values derived from Welch Two Sample *t*-tests comparing the cell membrane localization of *gfp::PH* and uniform *set-3*. *P* value legend: *<0.05.

Development • Supplementary information

Table S1. Worm strains used in study

Click here to download Table S1

Table S2. *E. coli* strains used in study.

Click here to download Table S2

Table S3. smFISH and smiFISH probe sets used in study.

Click here to download Table S3

Table S4. Total list of FERM and PH-like domain containing genes at the 2-cell stage based on scRNA-Seq.

Click here to download Table S4
