# Supplementary material for: The ERM-1 membrane-binding domain directs erm-1 mRNA localization to the plasma membrane in the C. elegans embryo
Source: Development. 2022 Nov 21;149(22):dev200930. doi: 10.1242/dev.200930 (PMC9793419; doi:10.1242/dev.200930)
Supplement: Supplementary file 1 [file develop-149-200930_review_history.pdf]

The ERM-1 membrane-binding domain directs *erm-1* mRNA localization to the plasma membrane in the *C. elegans* embryo

Lindsay P. Winkenbach, Dylan M. Parker, Robert T. P. Williams and Erin Osborne Nishimura
DOI: 10.1242/dev.200930

Editor: Swathi Arur

Review timeline
Original submission: 13 May 2022
Editorial decision: 14 June 2022
First revision received: 23 September 2022
Accepted: 10 October 2022

Original submission

First decision letter

MS ID#: DEVELOP/2022/200930

MS TITLE: Translation of the ERM-1 membrane-binding domain directs *erm-1* mRNA localization to the plasma membrane in the *C. elegans* embryo

AUTHORS: Lindsay P Winkenbach, Dylan M Parker, Robert TP Williams, and Erin Osborne Nishimura

I have now received all the referees' reports on the above manuscript, and have reached a decision. As you will see, the referees express considerable interest in your work, and suggest several edits, most of which are textual, which will need to be addressed before we can consider publication. Specially the reviewers state that from the experiments conducted, it is unclear whether or not localized translation is important and why so many mRNAs are not localizing. With specific regard to Figure 4, all three reviewers ask for a clarification on why the pattern of mRNA localization does not overlapp with the pattern of protein localization. If you are able to revise the manuscript along the lines suggested, some of which may require experimentation, I will be happy receive a revised version of the manuscript. Your revised paper will be re-reviewed by one or more of the original referees, and acceptance of your manuscript will depend on your addressing satisfactorily the reviewers' major concerns.

Please attend to all of the reviewers' comments and ensure that you clearly highlight all changes made in the revised manuscript. Please avoid using 'Tracked changes' in Word files as these are lost in PDF conversion. I should be grateful if you would also provide a point-by-point response detailing how you have dealt with the points raised by the reviewers in the 'Response to Reviewers' box. If you do not agree with any of their criticisms or suggestions please explain clearly why this is so.

Reviewer 1

Advance summary and potential significance to field

The work extends the broadening list of genes with translation dependence for mRNA localization, and defines the critical protein sequences for localization.

© 2022. Published by The Company of Biologists under the terms of the Creative Commons Attribution License (https://creativecommons.org/licenses/by/4.0/).

Comments for the author

General
Only about 20% of the RNA is localized. Please comment on why the % is so low. Do they think the localization is functionally important? Highly dynamic? The section in the Discussion is great, perhaps expand with a few more thoughts.

In the main text is stated that the FERM domain represents the peptide signal to localize ERM-1 RNC (including its corresponding mRNA) (lines 269-271). If this is the case, a sufficiency experiment would be terrific to test this statement.

In the early embryo, wild-type erm-1 mRNA generally co-localizes with its encoded protein (Fig. 4B – left panel). As far as I can see from your images, this is not the case at later stages (Fig. 4B – right panel), where the protein is enriched apically, but the mRNA is localized all over the membrane. How do you envision such differences? A comment in the discussion would be useful.

Figures:
Figure 1C, please include a graph of the ifg-1 RNAi with and without GFP visible.
The combined data that are shown are not convincing, but perhaps splitting the data into the two categories will be clearer.
Figure 2 could the authors add quantitation for # RNAs are localized with cycloheximide vs buffer control and RNAi control (e.g. similar to quantitation in 1D and 3D). Does CX lead to more localization, given that ribosomes are stalled and sitting around?
Fig. 4B: provide quantitation (e.g., like in Fig. 3D) would be nice, even though the text says there is no localization. Ie how many embryos examined. How much localization for the others.
Fig. 5A: add “merge” to the panel where only “membrane” is written (e.g., like in Fig. 1A)
Fig. 6: remove “A” which is not in the text. In the very first RNC, it seems like the peptide binding protein binds either the ribosome or the very N-terminus of the nascent peptide (which is not the case in either scenario). For clarity, I would recommend to remove the peptide binding protein cartoon from the very first RNC, and make it appear only in the second RNC where the PIP2 part of the FERM domain is shown.
Figure 5 please add quantitation.

Text:
Lines 154-155: “(…) erm-1 mRNA localization to the plasma membrane depended on translation-initiation for establishment and maintenance.” I am not sure that from the data we can talk about “maintenance”.
Lines 252-253: “In these embryos, the ERM-1[T544A] protein displayed increased basolateral localization in the intestinal lumen (…)” Why is this information important? Also this is not evident the change in localization in Fig. 4B (zoom-in, maybe?).
Line 266: “(…) and protein localization was reduced.” As above.
Lines 286-287: Fig. S6 and S7 come before S4 and S5. Please, correct the order.
Lines 318-319: “(…) F07C6.4 mRNA had uniform distribution (Fig. 5A) (…)” Not Fig. 5A, but S4A.
Line 321: “(…) localized to adherens junctions (…)”. It looks to me like it is enriched at the whole apical membrane, and not specifically at the adherens junctions.
Lines 324-326: “(…) where ANI-2 functions in maintaining the structure of the syncytial compartment of germline cytoplasm at the membrane (Maddox et al., 2005).” ANI-2 functions only in the adult gonad and not at embryonic stage under study. I am not sure about the connection with the mRNA localization this embryonic stage.

Supplemental figures:
Fig. S3A: I have hard times in understanding the nomenclature of the genotypes in the three panels. Also reading the figure legend does not help. In my head I read the following:
erm-1[4KN]::gfp / - : heterozygous, one allele is erm-1[4KN]::gfp and the other one a mutation
erm-1[4KN]::gfp / + : heterozygous, one allele is erm-1[4KN]::gfp and the other one wild-type erm-1[4KN]::gfp : homozygous for erm-1[4KN]::gfp erm-1[4KN]::gfp is a CRISPR line (BOX233: erm-1(mib21[erm-1[4KN]::GFP]) / dpy-5(e61) unc-29(e403) I – please revise nomenclatures in Table S1), these genotypes cannot be segregated by this sole line.
Furthermore, in erm-1[4KN]::gfp / - , is erm-1 mRNA real signal or background?

© 2022. Published by The Company of Biologists under the terms of the Creative Commons Attribution License (https://creativecommons.org/licenses/by/4.0/).

Fig. S4: seven mRNAs have been described to display a “clustered subcellular patterning in the posterior cell” (line 292). Among those, I am having hard times in seeing any pattern for mrck-1, wsp-1, and exoc-8.
Although ani-1 and frm-4 mRNAs membrane localization is clear, a co-stain for a membrane marker for frm-7 would be useful.
Fig. S5: in B, I do not see an enrichment at the plasma membrane, more like an exclusion from the nuclei. The cells at this embryonic stage are rather small and the co-localization of the mRNA with the plasma membrane seems to me an overstatement. In C, I do not see an enrichment of let-502 mRNA at the adherens junctions, but more widely at the apical membrane of epidermal cells.

Reviewer 2

Advance summary and potential significance to field

In this paper L. Winkenbach and collegues describe their works towards understanding localized mRNA expression of erm-1 in the C. elegans early embryo.
They have combined beautiful sm(i)Fish imaging with genetic manipulations, drug and heat shock treatments and extend their work beyond erm-1 when addressing mRNA localization of other FERM domain containing proteins. The role of mRNA localization in accurate gene expression control has received a lot of attention over the last years, and this paper fits the current interest in the field Overall the paper was very well written, the experiments are well thought-out and experimental details were in most cases explained very clear. The conclusions seemed to be sound and supported by several lines of experimental evidence. Therefore, I think that with some minor revisions (see below) the paper is suited for publication in Development

Comments for the author

Below I will list some comments, which may help to further improve the manuscript. Most comments could be addressed by changing the text or add some clarifications.

The main message from the manuscript is that translation of erm-1 mRNA leads to the specific localization of the mRNA. This localization specifically depends on the translation of the FERM domain, which can interact with the membrane. While several lines of evidence make the model very likely, it is not excluded that other molecular mechanism are in place as well. Therefore, I suggest that the authors also highlight more specifically what would be required to fully test and understand their model

Most importantly, the model predicts that translated mRNAs are located at the membrane, while mRNAs which are not undergoing translation are not at the membrane. The most important weakness of the paper is that the authors don’t have any direct measurements for the translation status of individual mRNA molecules, and therefore the conclusions so far are based on more indirect measurements. I realize that such direct, single molecule imaging of translation are very challenging and this is probably beyond the scope of this paper. However, it would be nice if the authors could reflect on this in the discussion.

In the discussion, the authors state that localized translation of the erm-1 mRNA could be possibly important for erm-1 function. As mutants that separate mRNA localization from protein localization are not available, there is no evidence that the localization of erm-1 mRNA is important. Moreover, it is unclear whether mRNA localization is an active process, or simply the consequence of mRNA diffusion where translated mRNAs randomly diffuse through the cytoplasm, and are captured at the membrane once the nascent peptide (encoding the FERM domain) interacts with the membrane is not clear. In my opinion the most likely model for mRNA localization is diffusion, where translated and non-translated mRNAs are freely diffusing through the cytoplasm. Upon translation, the FERM domain will be synthesized first, which will (once it finds the membrane, due to random diffusion) interact with the membrane, while the rest of the mRNA is still undergoing translation. As a consequence translated mRNAs will accumulate at the membrane, resulting in a specific localization pattern of the mRNA (however, this doesn’t serve any function). The authors seem to favor a model in which 1) the localized translation is important for erm-1 function and 2) there is active transport of translating mRNA molecules toward the membrane. While this could be possible,

© 2022. Published by The Company of Biologists under the terms of the Creative Commons Attribution License (https://creativecommons.org/licenses/by/4.0/).

there is no specific evidence for this and I would suggest that the authors highlight a bit more that so far there is no evidence that localized translation of erm-1 mRNA is important and that the observed mRNA localization pattern could be caused by random diffusion, rather than active transport.

In addition I have a few more specific comments regarding the manuscript, which I will list below
With regard to Figure 1:
• The figures and quantification show that there is an enrichment of erm-1 mRNA at the plasma membrane, which is lost upon ifg-1 knockdown or HS. However the figures also show that there are many mRNA molecules which do not localize to the membrane. Do the authors assume that these mRNAs are not translated, or are they translated, but don't localized to the membrane for any other reason.
Could the authors comment on this in the paper
• The authors use ifg-1 RNAi to knockdown translation, and measure los of translation based on the MODPEST::GFP::H2B construct. However, if the ifg-1 knockdown would be efficient and translation would have been stopped, these embryo's would not develop, as the novo protein synthesis is required for the oocyte to embryo transition. Therefore, in this experiment, translation could not have been blocked completely. So, if translation is not blocked completely why do we observe a complete loss of the localization pattern. If, for example translation has been reduced to 50%, we would only expect a 50% reduction of the number of mRNAs at the plasma membrane, but not a 100%. Could the authors comment in the paper on 1) the levels of ifg-1 knockdown 2) how well translation is inhibited in this assay and 3) why, if translation is not fully inhibited we don't see any enrichment of mRNAs at the plasma membrane anymore.

With regard to Figure 2:
• Addition of CHX doesn't change the localization pattern of erm-1 mRNA.
Could they authors provide data that show that CHX has entered the embryo and that ribosomes are stalled?

With regard to Figure 3:
• Based on the methods section it wasn't totally clear to me how the synon. strain was created an which specific sequence has been used. Did the authors do anything to prevent germline silencing? Could this be clarified in the methods section?

With regard to Figure 4:
• In this Figure mRNA localization was quantified in different mutants.
Since these strains are not homozygous viable, I was wondering how the authors ensure that the imaging was performed on homozygous mutants. How are sufficient number of embryo's obtained for smFish and how is the contribution of WT maternal product excluded. I fully trust the authors on their analysis, but to me it is not totally clear how this experiment was performed. Thank you very much for clarifying this.
• In Figure 4 the authors look in 4-cell state embryo's and in 2.5 fold embryo's. In WT erm-1 embryo's and in erm-1 [t544A] embryo's the authors observe a localization of the erm-1 mRNA to the plasma membrane, which is lost in the 4KN mutant. Important however, in the 2.5 cell stage embryo's, the mRNA localization pattern doesn't overlap with the protein localization (in WT and [t544A]), as mRNA seem to localize to all membranes (and maybe even more to baso-lateral than to the apical domain), while the protein localized apically.
These data suggest that the model, where mRNAs localize because of protein localization can not be true in these developmental stages. Could the authors explain in the paper why they think that the mRNA and protein localization pattern don't overlap here and how this fits in to their model? In line with this: how is the overlap between mRNA localization and protein localization of erm-1 in other developmental stages (larvae) and tissues. Are there tissues and stages where the mRNA is not localized and can this be explained by translational status (since erm-1 is a very stable protein)

With regard to Figure 5:
• When erm-1 is translated, the FERM domain will exit the ribosome tunnel first. The FERM domain can than interact with the membrane and drag the mRNA towards the mRNA. In case of ani-1 the PH-like domain (which should cause the interaction with the membrane) is located at the C-terminal. As a consequence very shortly after making the PH-like domain, the nascent protein will be released, and no longer be together with the mRNA. Therefore, the model where the protein

© 2022. Published by The Company of Biologists under the terms of the Creative Commons Attribution License (https://creativecommons.org/licenses/by/4.0/).

domain drags the mRNA to the membrane is less likely to be true for this protein. Could the authors comment on this in the discussion and explain how this fits into their model?

With regard to Figure 6:
From the text or the Figure legend, it is not clear to what the yellow ‘peptide binding protein’ is and what the role of the protein is. Could the authors clarify this.

Reviewer 3

Advance summary and potential significance to field

In the current study, Winkenabach et al discover the essential role of the membrane-binding (FERM) domain in membrane-associated localization of *erm-1* mRNA, and probably, of multiple other mRNAs encoding FERM domain, in *C. elegans* embryo. Using multiple approaches that perturb translation, they first showed that *erm-1 mRNA localization depends on translation. By re-coding the erm-1* mRNA coding sequence without altering amino acid sequence, they further confirmed that *erm-1* mRNA localization depends on protein sequence but not mRNA sequence. Next they mapped the FERM domain, but not the phosphorylation site, involved in mRNA localization. Then they identified three more membrane localized mRNAs in early embryos and 9 additional transcripts associated with membrane at later stages through smiFISH screen of 17 FERM and/or PH domain(s) encoding genes.

mRNA localization is a prevalent rule regulating gene expression in many cell types. Recently, the studies on mRNA localization and its mechanism and function have attracted a lot of attention. Specifically, Tocchini et al. (Development 2021) showed translation-dependent *dlg-1* mRNA localization. Given the distinct focus of proteins and developmental stages as well as the approaches used of the two studies, this would be a great addition to the literature. The analysis on localization pattern of previously uncharacterized mRNAs will serve as a resource to the biologist in this field and be of interest to the general readership of Development.

Comments for the author

Main comments:
- It is very nice that the authors used multiple approaches to demonstrate that membrane localization of *erm-1* mRNA requires translation initiation and intact association of nascent peptide and ribosomes. The authors then used an elegant sequence scrambling method to show that *erm-1* mRNA localization does not depend on mRNA sequence and further narrowed down to the membrane binding domain by testing different mutants. Both individual parts are solid and convincing, however, the combination of the two parts is not enough to conclude that *erm-1 mRNA* localization depends on the translation of ERM-1 protein, much less on the translation of membrane -binding domain. It would be more convincing that if the authors can show specifically that ERM-1 translation is required and/or visualizing translation of ERM-1 is associated with mRNA localization. If this cannot be shown, the title should be revised to reflect the data accurately.
- Figure 1 and 2: A negative mRNA control whose localization is known to be translation-independent should be included. If, as expected, the treatment leads to unaltered localization of the control mRNA, it would help to convince reader that the effects on *erm-1* mRNA localization is derived from the translation-dependent nature of the mRNA, but not secondary to the changes of other factors upon translation perturbation.
- Heat shock can affect many things in addition to translation inhibition. It would be good to use some kind of readout to confirm translation inhibition together with *erm-1* mRNA mislocalization.
- For Figure 4B at 2.5 stage, wild type *erm-1* and *erm-1[T544A]* mRNAs are still localized to the plasma membranes, whereas *erm-1[4KN]* mRNAs are cytoplasmic. One could easily assume the membrane-associated mRNA localization at this stage still depends on the membrane-binding domain, and translation as well (similar to the conclusions for 4-cell stage embryo). Therefore, one would expect to see partial colocalization of protein and mRNA at plasma membrane as observed in 4-cell stage embryo. However, at this stage proteins are enriched in apical membrane, but not co-localizing with mRNA. I am wondering how the authors interpret this part of results. Does it mean the translational dependence of *erm-1* is stage specific? Or, does it mean colocalization of mRNA

© 2022. Published by The Company of Biologists under the terms of the Creative Commons Attribution License (https://creativecommons.org/licenses/by/4.0/).

with at least some of the proteins is not the prerequisite of translation-dependent mRNA localization? The authors should add interpretation and discussion into the result session.

Minor comments:
- Page 6 line 135: I think a detailed description of the destabilized-GFP strain would help.
- P13 line 326-327: 9 of the surveyed transcripts…, actually there are 10 genes listed in the bracket.
- P18 line 451-452: “to induce bursting from to” – something seems to be missing here.
- P17 line 412: *Drosophila* should be italic.
- Figure 2: number of embryos/animals should be indicated in the figure legend or figure.
- Figure 4: for the quantification, number of cells/animals should be indicated.
- Figure S2: the labels in the figure are not consistent with the description of figure legend.
- Figure S3: the use of *erm-1[4KN]::gfp/-* is confusing, because the worms carrying *erm-1::gfp* can also be considered as *erm-1[4KN]::gfp* null. From phenotype, it looks like N2 animals. To me, N2 is more straightforward. Alternatively, *erm-1[4KN]::gfp/-* should be defined explicitly first.
- Figure S4: I am confused about whether the anterior- or posterior-enrichment is from this study or previous study. If from this study, it is hard to appreciate the anterior enrichment for all the transcripts in panel A except *ani-1*. Purely based on the pictures shown here, *frm-4* transcripts look a bit like anterior-enriched, *Y41E3.7* posterior enriched and *exoc-8* more uniform.

First revision

Author response to reviewers' comments

Reviewer 1 Advance Summary and Potential Significance to Field...

The work extends the broadening list of genes with translation dependence for mRNA localization, and defines the critical protein sequences for localization.

Reviewer 1 Comments for the Author...

General

Only about 20% of the RNA is localized. Please comment on why the % is so low. Do they think the localization is functionally important? Highly dynamic? The section in the Discussion is great, perhaps expand with a few more thoughts.

Response 1.1. We are unclear what the **20 % statistic** references. Is the reviewer stating that only 20 % of *erm-1* mRNA transcripts are localized to the membrane? This was not specifically quantified in our manuscript, though at the 4-cell stage of development roughly 40 % of *erm-1* mRNA localizes to within 10% normalized distance of the membrane. We have added this statistic to the Discussion Section (Line 490-491) and **added a discussion sub-section, “What is the status of the cytosolic population of erm-1 mRNA?”** (Lines 489 – 499) Briefly, we speculate that he plasma membrane localized fraction is undergoing translation and the cytoplasmic fraction is not. We suggest that nascent chain tracking technologies could be used to test this hypothesis.

Response 1.2. Another possibility is that the reviewer is referring to the fact that **20 %** of the PH- and FERM-containing genes yielded localized transcripts at the 4-cell stage (3 of 17 that were surveyed). Is this the meaning? We do not know the function of these localization patterns, but the observation that multiple FERM- and PH- containing transcripts are also localized points to a greater probability that this is a general phenomenon. To address the reviewer’s concerns, we have expanded the Results section to state this idea more clearly. (Lines 391 – 395)

Response 1.3. We do not know whether localization of *erm-1* mRNA itself is **functional** nor do we know the **dynamics**. To address the reviewer’s concerns and to clarify the manuscript, we have added a **new discussion sub-section, “What is the function of *erm-1* mRNA localization?”** (Lines

© 2022. Published by The Company of Biologists under the terms of the Creative Commons Attribution License (https://creativecommons.org/licenses/by/4.0/).

423 – 461) Briefly, we outline three models: 1) *erm-1* mRNA localization serves to deposit ERM-1 at discrete locations that influences morphology and cell biology, 2) ERM-1 is locally translated to guard against negative intermolecular interactions, or 3) *erm-1* mRNA localization is the passive result of ERM-1 proteins that localize during translation. Reformatting the discussion in this way has greatly improved it and we thank the reviewer for raising this point. We have also added **a new version of Figure 6** that incorporates this idea.

Response 1.4. To address dynamics, we better delineated in the discussion how nascent chain tracking technology and fluorescence assay to detect ribosome interactions with mRNA (FLARIM) could be well suited to address *erm-1* mRNA localization and translation dynamics in future works. (Lines 482 – 487; 496 – 499)

In the main text is stated that the FERM domain represents the peptide signal to localize ERM-1 RNC (including its corresponding mRNA) (lines 269-271). If this is the case, a sufficiency experiment would be terrific to test this statement.

Response 2.1 We thank the reviewer for this suggestion and agree that a **sufficiency experiment** would strengthen the paper. To address this, we assessed whether a PH domain in the membrane marker strain (GFP::PH) is sufficient to direct *gfp* mRNA to the membrane, and we found that it did by 1.3-fold enrichment compared to the uniform control *set-3* (New Figure S9). This worm strain (*mex-5p::GFP::PH::tbb-2 3'UTR*) is a single copy transgenic knock-in using the PH domain from human phospholipase C-delta-1. **Added content: Figure S9 (Lines 373 – 387)**

Response 2.2. In addition, we tried **two other approaches** to test the sufficiency of the PH domain in directing mRNA to the plasma membrane but were unsuccessful. 1) We fused the ERM-1 PH domain to GFP and introduced it into worms as an extrachromosomal array. Sadly, this strain silenced. 2) We ordered a single-copy MosSCI insertion of the ERM PH domain fused to GFP from InVivo Scientifics. However, the construction of that strain was delayed. In lieu of these approaches, we hope the GFP::PH experiment added in Figure S9 will satisfy the reviewer that PH domains can direct mRNA localization.

In the early embryo, wild-type erm-1 mRNA generally co-localizes with its encoded protein (Fig. 4B – left panel). As far as I can see from your images, this is not the case at later stages (Fig. 4B – right panel), where the protein is enriched apically, but the mRNA is localized all over the membrane. How do you envision such differences? A comment in the discussion would be useful.

Response 3.1. We thank the reviewer for raising this idea as it has stimulated discussions that have improved the paper. The major difference is that in non-polarized cells ERM-1 localizes to plasma membranes via the FERM domain, but in the polarized cells of the intestine, ERM-1 localization occurs via a **2-step process**. The first step localizes ERM-1 to plasma membrane (FERM domain), and the second step moves it to the apical face by virtue of the C-ERMAD domain (Ramhalo et al., 2020). Motivated by the reviewer's comments, we thought more deeply about our observations and determined that the fact that *erm-1* mRNA only localizes to the PM never concentrating at intestinal apical membranes (as the protein does) suggests that the second step is likely occurring post-translation.

To explain this insight more clearly, we have **rewritten the Results section "*erm-1* mRNA and ERM-1 protein localization require the ERM-1 PIP2 membrane-binding region"** (Lines 244 – 296). We have **added a discussion sub-section "How do *erm-1* mRNA and ERM-1 protein localize in polarized cells?" (Lines 501 – 510)**. We have added a new explanatory model figure: **new Figure 4D**.

Figures:

Figure 1C, please include a graph of the ifg-1 RNAi with and without GFP visible. The combined data that are shown are not convincing, but perhaps splitting the data into the two categories will be clearer.

Response 4.1. Our apologies, but we aren't clear what a graph of *ifg-1* RNAi would quantify. For each embryo, we have categorically binned the embryos for 1) localized *erm-1* mRNA or 2)

© 2022. Published by The Company of Biologists under the terms of the Creative Commons Attribution License (https://creativecommons.org/licenses/by/4.0/).

dispersed *erm-1* mRNA. For each of those embryos, we know the quantitated GFP intensity of the reporter. Therefore, it made more sense to graph a numerical value against a categorical value.

Figure 2 could the authors add quantitation for # RNAs are localized with cycloheximide vs buffer control and RNAi control (e.g. similar to quantitation in 1D and 3D). Does CX lead to more localization, given that ribosomes are stalled and sitting around?

Response 5.1. We thank the reviewer for this suggestion. We are also interested in the question of whether cycloheximide leads to increased localization. However, due to technical limitations of the experimental set up we were unable to perform the quantification needed to address this. All embryos for the PERM RNAi followed by drug treatment were hand-dissected and then mounted onto slides by hand, unlike our other protocols that use in-tube smFISH assays. Under the hand-dissection conditions, it has been difficult to obtain samples with sufficiently low background in the FITC channel for us to image GFP reliably. Our lab is currently working to improve this approach so quantification will be attainable in the future.

Fig. 4B: provide quantitation (e.g., like in Fig. 3D) would be nice, even though the text says there is no localization. Ie how many embryos examined. How much localization for the others.

Response 6.1. We thank the reviewer for this suggestion, but we were unable to quantify that metric in the 4KN mutant as the GFP membrane marker also became mis-localized. Without a membrane marker, our quantification algorithm fails. Similarly, the membrane marker in the T544A mutant is more diffuse, preventing quantification. In lieu of this, we reported the number embryos observed with the reported patterns of localization (in the Fig 4 legend) and the total number of mRNA molecules in each mutant versus wild type (Fig 4B).

Fig. 5A: add “merge” to the panel where only “membrane” is written (e.g., like in Fig. 1A)

Response 7.1. We have made this change.

Fig. 6: remove “A” which is not in the text. In the very first RNC, it seems like the peptide binding protein binds either the ribosome or the very N-terminus of the nascent peptide (which is not the case in either scenario). For clarity, I would recommend to remove the peptide binding protein cartoon from the very first RNC, and make it appear only in the second RNC where the PIP2 part of the FERM domain is shown.

Response 8.1. We have made this change.

Figure 5 please add quantitation.

Response 9.1. We have **added quantitation** of membrane enrichment for *ani-1* and *frm-4* mRNAs at the 4-cell stage (**new Fig. 5B**) and for *frm-7* at the 2-cell and 4-cell stages (**new Fig. S7C**). Also, we added quantitation of the number of RNA clusters of *unc-112* and *ani-2* mRNAs based on their overlap with the GLH-1::GFP P granule marker (**new Fig. 5C**).

Lines 154-155: “(…) erm-1 mRNA localization to the plasma membrane depended on translation-initiation for establishment and maintenance.” I am not sure that from the data we can talk about “maintenance”.

Response 10.1. We agree with the reviewer and have **removed the word maintenance** from the results section (Lines 159 – 160)

Lines 252-253: “In these embryos, the ERM-1[T544A] protein displayed increased basolateral localization in the intestinal lumen (…)” Why is this information important? Also this is not evident the change in localization in Fig. 4B (zoom-in, maybe?).

Addressed in Response 3.1

Line 266: “(…) and protein localization was reduced.” As above.

© 2022. Published by The Company of Biologists under the terms of the Creative Commons Attribution License (https://creativecommons.org/licenses/by/4.0/).

Addressed in Response 3.1

Lines 286-287: Fig. S6 and S7 come before S4 and S5. Please, correct the order.

Response 11.1. We have corrected this.

Lines 318-319: "(…) F07C6.4 mRNA had uniform distribution (Fig. 5A) (…)" Not Fig. 5A, but S4A.

Response 12.1. We have corrected this.

Line 321: "(…) localized to adherens junctions (…)". It looks to me like it is enriched at the whole apical membrane, and not specifically at the adherens junctions.

Response 13.1. We have corrected this (Lines 356 - 357).

Lines 324-326: "(…) where ANI-2 functions in maintaining the structure of the syncytial compartment of germline cytoplasm at the membrane (Maddox et al., 2005)." ANI-2 functions only in the adult gonad and not at embryonic stage under study. I am not sure about the connection with the mRNA localization this embryonic stage.

Response 14.1. We thank the reviewer for this comment. We have **updated the reference** for ANI-2 localization by including a publication by Amini et al (Lines 359 - 361). By GFP tagging ANI-2, Amini et al showed that ANI-2 is enriched at the rachis bridge of germ cells from the onset of germ cell specification.

Supplemental figures:

Fig. S3A: I have hard times in understanding the nomenclature of the genotypes in the three panels. Also reading the figure legend does not help. In my head I read the following:
erm-1[4KN]::gfp / - : heterozygous, one allele is erm-1[4KN]::gfp and the other one a mutation
erm-1[4KN]::gfp / + : heterozygous, one allele is erm-1[4KN]::gfp and the other one wild-type erm-1[4KN]::gfp : homozygous for erm-1[4KN]::gfp
erm-1[4KN]::gfp is a CRISPR line (BOX233: erm-1(mib21[erm-1[4KN]::GFP]) / dpy- 5(e61) unc-29(e403) I – please revise nomenclatures in Table S1), these genotypes cannot be segregated by this sole line. Furthermore, in erm-1[4KN]::gfp / - , is erm-1 mRNA real signal or background?

Response 15.1. The nomenclature was not only confusing but was also mislabeled adding to the confusion. We have **corrected and updated the nomenclature** in Supplemental Figure S3 and in the supplemental figure legends…

- *erm-1 / erm-1* – homozygous wild-type for the erm-1 allele
- *erm-1[4KN]::gfp / erm-1* – a heterozygote containing one copy of the *erm-1[KN]::gfp* allele and one of the wild-type *erm-1* allele.
- *erm-1[4KN]::gfp / erm-1[4KN]::gfp* – homozygous for the *erm-1[4KN]::gfp* allele.

Fig. S4: seven mRNAs have been described to display a "clustered subcellular patterning in the posterior cell" (line 292). Among those, I am having hard times in seeing any pattern for mrck-1, wsp-1, and exoc-8. Although ani-1 and frm-4 mRNAs membrane localization is clear, a co-stain for a membrane marker for frm-7 would be useful.

Response 16.1. We thank the reviewer for the comment and see how the representative images for *mrck-1, wsp-1,* and *exoc-8* are difficult to interpret. We have reexamined all early embryos for these three transcripts and observe persistent clustering in *wsp-1* and *exoc-8* but not for *mrck-1*. We agree *mrck-1* looks uniformly distributed and have **amended Table 1**.

Response 16.2. For *frm-7*, we agree the localization in 4-cell embryos is unclear. However, the localization in the 2-cell stage is. We have **added more figures and additional quantification *frm-7* (new Fig S7).**

© 2022. Published by The Company of Biologists under the terms of the Creative Commons Attribution License (https://creativecommons.org/licenses/by/4.0/).

Fig. S5: in B, I do not see an enrichment at the plasma membrane, more like an exclusion from the nuclei. The cells at this embryonic stage are rather small and the co-localization of the mRNA with the plasma membrane seems to me an overstatement. In C, I do not see an enrichment of let-502 mRNA at the adherens junctions, but more widely at the apical membrane of epidermal cells.

Response 17.1. We agree it is difficult to interpret localization patterns for *F07C6.4* at this stage for the reasons stated. We have **amended language** in the results section to reflect this difficulty (Line 352). In terms of *let-502*, we agree assigning localization as the apical membrane of epidermal cells is more accurate and we have **amended the text accordingly (Lines 356 - 357).**

We have added a section explaining the limitations of interpreting these localization patterns (Lines 367 – 371).

Reviewer 2 Advance Summary and Potential Significance to Field...

In this paper L. Winkenbach and collegues describe their works towards understanding localized mRNA expression of erm-1 in the C. elegans early embryo. They have combined beautiful sm(i)Fish imaging with genetic manipulations, drug and heat shock treatments and extend their work beyond erm-1 when addressing mRNA localization of other FERM domain containing proteins. The role of mRNA localization in accurate gene expression control has received a lot of attention over the last years, and this paper fits the current interest in the field. Overall the paper was very well written, the experiments are well thought-out and experimental details were in most cases explained very clear. The conclusions seemed to be sound and supported by several lines of experimental evidence. Therefore, I think that with some minor revisions (see below) the paper is suited for publication in Development.

Reviewer 2 Comments for the Author...

Below I will list some comments, which may help to further improve the manuscript. Most comments could be addressed by changing the text or add some clarifications.

The main message from the manuscript is that translation of erm-1 mRNA leads to the specific localization of the mRNA. This localization specifically depends on the translation of the FERM domain, which can interact with the membrane. While several lines of evidence make the model very likely, it is not excluded that other molecular mechanism are in place as well. Therefore, I suggest that the authors also highlight more specifically what would be required to fully test and understand their model

Response 18.1. We thank the reviewer for noticing this. To address their concerns, we have **expanded the discussion to include a sub-section titled "What pathways localize *erm-1* to the plasma membrane in the early embryo?" (Lines 463 - 487).** Briefly, the new section outlines alternative mechanisms that could localize *erm-1* (passive diffusion v. active trafficking), possible factors that may be involved in localization, whether ongoing translation or a pause in translation may be occurring, and how nascent chain tracking could be used to address these questions.

Most importantly, the model predicts that translated mRNAs are located at the membrane, while mRNAs which are not undergoing translation are not at the membrane. The most important weakness of the paper is that the authors don't have any direct measurements for the translation status of individual mRNA molecules, and therefore the conclusions so far are based on more indirect measurements. I realize that such direct, single molecule imaging of translation are very challenging and this is probably beyond the scope of this paper. However, it would be nice if the authors could reflect on this in the discussion.

Addressed in Response 1.1

In the discussion, the authors state that localized translation of the erm-1 mRNA could be possibly important for erm-1 function. As mutants that separate mRNA localization from protein localization are not available, there is no evidence that the localization of erm-1 mRNA is important. Moreover, it is unclear whether mRNA localization is an active process, or simply the consequence of mRNA

© 2022. Published by The Company of Biologists under the terms of the Creative Commons Attribution License (https://creativecommons.org/licenses/by/4.0/).

diffusion where translated mRNAs randomly diffuse through the cytoplasm, and are captured at the membrane once the nascent peptide (encoding the FERM domain) interacts with the membrane is not clear. In my opinion the most likely model for mRNA localization is diffusion, where translated and non-translated mRNAs are freely diffusing through the cytoplasm. Upon translation, the FERM domain will be synthesized first, which will (once it finds the membrane, due to random diffusion) interact with the membrane, while the rest of the mRNA is still undergoing translation. As a consequence, translated mRNAs will accumulate at the membrane, resulting in a specific localization pattern of the mRNA (however, this doesn't serve any function). The authors seem to favor a model in which 1) the localized translation is important for erm-1 function and 2) there is active transport of translating mRNA molecules toward the membrane. While this could be possible, there is no specific evidence for this and I would suggest that the authors highlight a bit more that so far there is no evidence that localized translation of erm-1 mRNA is important and that the observed mRNA localization pattern could be caused by random diffusion, rather than active transport.

Response 19.1. Thank you for raising this point as it highlights our oversight. Yes, we originally were agnostic whether localization of *erm-1* mRNA occurred through passive diffusion (coupled with anchoring) or active transport. However, a recent study that used live-imaging (PP7-PCP) to report *erm-1* mRNA movement in larval worms observed that their movements as rapid, directional, and dynein motor (DHC-1) dependent, suggesting *erm-1* localization requires active cytoskeletal trafficking (Li et al., 2021). We **have amended our discussion to include this information (Lines 471 - 480).**

In addition I have a few more specific comments regarding the manuscript, which I will list below.

With regard to Figure 1: The figures and quantification show that there is an enrichment of erm-1 mRNA at the plasma membrane, which is lost upon ifg-1 knockdown or HS. However, the figures also show that there are many mRNA molecules which do not localize to the membrane. Do the authors assume that these mRNAs are not translated, or are they translated, but don't localized to the membrane for any other reason. Could the authors comment on this in the paper

See Response 1.1.

With regard to Figure 1: The authors use ifg-1 RNAi to knockdown translation, and measure los of translation based on the MODPEST::GFP::H2B construct. However, if the ifg-1 knockdown would be efficient and translation would have been stopped, these embryo's would not develop, as the novo protein synthesis is required for the oocyte to embryo transition. Therefore, in this experiment, translation could not have been blocked completely. So, if translation is not blocked completely, why do we observe a complete loss of the localization pattern. If, for example translation has been reduced to 50%, we would only expect a 50% reduction of the number of mRNAs at the plasma membrane, but not a 100%. Could the authors comment in the paper on 1) the levels of ifg-1 knockdown 2) how well translation is inhibited in this assay and 3) why, if translation is not fully inhibited we don't see any enrichment of mRNAs at the plasma membrane anymore.

Response 20.1 We thank the reviewer for this comment and appreciate the attention to detail. We optimized the timing *ifg-1* RNAi to allow for roughly 50 % of worms at the 4-cell stage to exhibit signs of translational inhibition and 50% to not (reported by MODPEST::GFP fluorescence). Most importantly, 100 % of these are embryonic lethal. To address the questions the reviewer raised, we have **amended the description of this experiment in the Results section (Lines 138 - 146) and in the Materials & Method section (Lines 548 - 549).**

With regard to Figure 2: Addition of CHX doesn't change the localization pattern of erm-1 mRNA. Could they authors provide data that show that CHX has entered the embryo and that ribosomes are stalled?

Response 21.1. We thank the reviewer for this comment. Both cycloheximide and puromycin treatment induced cell cycle arrest. We have **added this in the Methods section**. The concentration and timing of drug treatment was based on the Chih-Yung Lee et al., 2020 paper in which the cell cycle arrest phenotype is documented.

© 2022. Published by The Company of Biologists under the terms of the Creative Commons Attribution License (https://creativecommons.org/licenses/by/4.0/).

With regard to Figure 3: Based on the methods section it wasn't totally clear to me how the synon. strain was created an which specific sequence has been used. Did the authors do anything to prevent germline silencing? Could this be clarified in the methods section?

Response 22.1. To clarify this, we have amended the Methods section describing the generation of the *erm-1* synonymous strain in a new subsection, **"Synonymous *erm-1* strain generation" (Lines 572 - 580)**

With regard to Figure 4: In this Figure mRNA localization was quantified in different mutants. Since these strains are not homozygous viable, I was wondering how the authors ensure that the imaging was performed on homozygous mutants. How are sufficient number of embryo's obtained for smFish and how is the contribution of WT maternal product excluded. I fully trust the authors on their analysis, but to me it is not totally clear how this experiment was performed. Thank you very much for clarifying this.

Response 23.1. We have expanded discussion in the results section referencing Figure S3A to describe how mutant embryos were identified by the number of *erm-1* and *gfp* mRNA molecules detected in *erm-1[4KN]::gfp* heterozygous offspring.

With regard to Figure 4: In Figure 4 the authors look in 4-cell state embryo's and in 2.5 fold embryo's. In WT erm-1 embryo's and in erm-1 [t544A] embryo's the authors observe a localization of the erm-1 mRNA to the plasma membrane, which is lost in the 4KN mutant. Important however, in the 2.5 cell stage embryo's, the mRNA localization pattern doesn't overlap with the protein localization (in WT and [t544A]), as mRNA seem to localize to all membranes (and maybe even more to baso-lateral than to the apical domain), while the protein localized apically. These data suggest that the model, where mRNAs localize because of protein localization can not be true in these developmental stages. Could the authors explain in the paper why they think that the mRNA and protein localization pattern don't overlap here and how this fits in to their model? In line with this: how is the overlap between mRNA localization and protein localization of erm-1 in other developmental stages (larvae) and tissues. Are there tissues and stages where the mRNA is not localized and can this be explained by translational status (since erm-1 is a very stable protein)

Addressed in Response 3.1

With regard to Figure 5: When erm-1 is translated, the FERM domain will exit the ribosome tunnel first. The FERM domain can than interact with the membrane and drag the mRNA towards the mRNA. In case of ani-1 the PH-like domain (which should cause the interaction with the membrane) is located at the C-terminal. As a consequence, very shortly after making the PH- like domain, the nascent protein will be released, and no longer be together with the mRNA. Therefore, the model where the protein domain drags the mRNA to the membrane is less likely to be true for this protein. Could the authors comment on this in the discussion and explain how this fits into their model?

Response 24.1. Yes, we had the initial expectation that FERM domains would necessarily need to be N-terminal to localize their encoding mRNA. And we agree that *ani-1* breaks this trend. We speculate that pauses in translation or successive rounds of translation occurring at the membrane could explain why we observe *ani-1* membrane localization so prominently. We have **added a clearer explanation of these ideas in the discussion (Lines 482 - 487).**

With regard to Figure 6: From the text or the Figure legend, it is not clear to what the yellow 'peptide binding protein' is and what the role of the protein is. Could the authors clarify this.

Response 25.1. We have removed this.

Reviewer 3 Advance Summary and Potential Significance to Field...

In the current study, Winkenabach et al discover the essential role of the membrane-binding (FERM) domain in membrane-associated localization of *erm-1* mRNA, and probably, of multiple other mRNAs encoding FERM domain, in *C. elegans* embryo. Using multiple approaches that perturb translation, they first showed that erm-1 mRNA localization depends on translation. By re-coding

© 2022. Published by The Company of Biologists under the terms of the Creative Commons Attribution License (https://creativecommons.org/licenses/by/4.0/).

the erm-1 mRNA coding sequence without altering amino acid sequence, they further confirmed that erm-1 mRNA localization depends on protein sequence but not mRNA sequence. Next they mapped the FERM domain, but not the phosphorylation site, involved in mRNA localization. Then they identified three more membrane localized mRNAs in early embryos and 9 additional transcripts associated with membrane at later stages through smiFISH screen of 17 FERM and/or PH domain(s) encoding genes.

mRNA localization is a prevalent rule regulating gene expression in many cell types. Recently, the studies on mRNA localization and its mechanism and function have attracted a lot of attention. Specifically, Tocchini et al. (Development 2021) showed translation-dependent dlg-1 mRNA localization. Given the distinct focus of proteins and developmental stages as well as the approaches used of the two studies, this would be a great addition to the literature. The analysis on localization pattern of previously uncharacterized mRNAs will serve as a resource to the biologist in this field and be of interest to the general readership of Development.

Reviewer 3 Comments for the Author...

Main comments:

It is very nice that the authors used multiple approaches to demonstrate that membrane localization of erm-1 mRNA requires translation initiation and intact association of nascent peptide and ribosomes. The authors then used an elegant sequence scrambling method to show that erm-1 mRNA localization does not depend on mRNA sequence and further narrowed down to the membrane binding domain by testing different mutants. Both individual parts are solid and convincing, however, the combination of the two parts is not enough to conclude that erm-1 mRNA localization depends on the translation of ERM-1 protein, much less on the translation of membrane -binding domain. It would be more convincing that if the authors can show specifically that ERM-1 translation is required and/or visualizing translation of ERM-1 is associated with mRNA localization. If this cannot be shown, the title should be revised to reflect the data accurately.

Response 26.1. We thank the reviewer for this careful feedback. We have revised the title (Line 6) and summary statement (Lines 29 – 30) accordingly. Future studies from our group will aim to address the translation status of *erm-1* more directly using nascent chain tracking technology. We have **included a description of this approach in the discussion (Lines 482 – 487; 496 - 499).**

Figure 1 and 2: A negative mRNA control whose localization is known to be translation-independent should be included. If, as expected, the treatment leads to unaltered localization of the control mRNA, it would help to convince reader that the effects on erm-1 mRNA localization is derived from the translation-dependent nature of the mRNA, but not secondary to the changes of other factors upon translation perturbation.

Response 27.1. We thank the reviewer for this comment as we wanted to include such a control. However, we were unable to find a suitable candidate. Many mRNA molecules localize to subcellular locales dependent on translation (*imb-2, dlg-1,* etc) and those that localize translational independently are already translationally repressed (*nos-2, cpg-2, chs-1,* etc). Lee et al., 2020 verified that inhibiting translation by heat shock does not change the localization patterning of *nos-2* to P granules. We have also heat shocked and drug treated many embryos and assessed the resulting localization of RNAs to P granules does not change (Parker et al., 2020). Again, because these transcripts are not undergoing active translation with or without the drugs, it is hard to rely upon these as controls for drug treatment.

Heat shock can affect many things in addition to translation inhibition. It would be good to use some kind of readout to confirm translation inhibition together with erm-1 mRNA mislocalization.

Response 28.1. We thank the reviewer for this comment. We acknowledge the difficulty in interpreting heat shock solely for its direct impact on translational inhibition. Luckily, a benefit of performing heat shock in early embryos that transcriptional responses are paused at this stage, removing some confounding indirect effects. The complex nature of heat shock is exactly why we chose to three methods for impeding translation (heat shock, drug treatment, and *ifg-1* RNAi).

© 2022. Published by The Company of Biologists under the terms of the Creative Commons Attribution License (https://creativecommons.org/licenses/by/4.0/).

Because all three treatments disrupted *erm-1* mRNA localization, we had greater confidence in concluding *erm-1* localization to the plasma membrane depends on translation.

We were also interested in using a complementary approach that would impede the translation of *erm-1* mRNA in a transcript-specific manner. Our original goal was to mutate the start codon of *erm-1*. However, because *erm-1* is annotated with two different start codons, this would have introduced mutations into some isoforms, complicated their interpretation.

To address the points the reviewer raises, we have **expanded the results section** to be more transparent on the limitations of this assay (Lines 159 – 160)

For Figure 4B at 2.5 stage, wild type erm-1 and erm-1[T544A] mRNAs are still localized to the plasma membranes, whereas erm-1[4KN] mRNAs are cytoplasmic. One could easily assume the membrane-associated mRNA localization at this stage still depends on the membrane-binding domain, and translation as well (similar to the conclusions for 4-cell stage embryo). Therefore, one would expect to see partial colocalization of protein and mRNA at plasma membrane as observed in 4-cell stage embryo. However, at this stage proteins are enriched in apical membrane, but not co-localizing with mRNA. I am wondering how the authors interpret this part of results. Does it mean the translational dependence of erm-1 is stage specific? Or, does it mean colocalization of mRNA with at least some of the proteins is not the prerequisite of translation-dependent mRNA localization? The authors should add interpretation and discussion into the result session.

Addressed in Response 3.1

Minor comments:

Page 6 line 135: I think a detailed description of the destabilized-GFP strain would help.

Response 29.1. We **have now included a description of the translation reporter strain. (Lines 138 - 146)**

P13 line 326-327: 9 of the surveyed transcripts…, actually there are 10 genes listed in the bracket.

Response 30.1. We have corrected this.

P18 line 451-452: “to induce bursting from to” – something seems to be missing here.

Response 31.1. We have corrected this.

P17 line 412: Drosophila should be italic.

Response 32.1. We have corrected this. Thank you.

Figure 2: number of embryos/animals should be indicated in the figure legend or figure.

Response 33.1. We have corrected this.

Figure 4: for the quantification, number of cells/animals should be indicated.

Response 34.1. We have corrected this.

Figure S2: the labels in the figure are not consistent with the description of figure legend.

Response 35.1. We have corrected this.

Figure S3: the use of erm-1[4KN]::gfp/- is confusing, because the worms carrying erm-1::gfp can also be considered as erm-1[4KN]::gfp null. From phenotype, it looks like N2 animals. To me, N2 is more straightforward. Alternatively, erm-1[4KN]::gfp/- should be defined explicitly first.

© 2022. Published by The Company of Biologists under the terms of the Creative Commons Attribution License (https://creativecommons.org/licenses/by/4.0/).

Addressed in Response 15.1.
Figure S4: I am confused about whether the anterior- or posterior-enrichment is from this study or previous study. If from this study, it is hard to appreciate the anterior enrichment for all the transcripts in panel A except ani-1. Purely based on the pictures shown here, frm-4 transcripts look a bit like anterior-enriched, Y41E3.7 posterior enriched and exoc-8 more uniform.

Response 36.1. We thank the reviewer for this comment. We have **amended the Results (Lines 309 - 316) and the Materials and Methods section (Lines 657 - 667)** to clearly reference Nishimura et al., 2015 and Tintori et al., 2016 that documented these transcripts as anterior enriched. Anterior enrichment may occur in other stags of embryogenesis from what we have imaged in our study.

Second decision letter

MS ID#: DEVELOP/2022/200930

MS TITLE: The ERM-1 membrane-binding domain directs *erm-1* mRNA localization to the plasma membrane in the *C. elegans* embryo

AUTHORS: Lindsay P Winkenbach, Dylan M Parker, Robert TP Williams, and Erin Osborne Nishimura
ARTICLE TYPE: Research Article

I am happy to tell you that your manuscript has been accepted for publication in Development, pending our standard ethics checks.

Reviewer 2

Advance summary and potential significance to field

I would like to thank the authors for their improvements to the manuscript. I specifically appreciate the effort made towards figure 4D, providing us with a nicely illustrated model.
I enjoyed reading the manuscript and I would publish the paper as it is.

Comments for the author

I would like to thank the authors for their improvements to the manuscript. I specifically appreciate the effort made towards figure 4D, providing us with a nicely illustrated model.
I enjoyed reading the manuscript and I would publish the paper as it is

Reviewer 3

Advance summary and potential significance to field

The revised manuscript by Winkenabach et al. has improved significantly, and the authors' response to my concerns are reasonable. The authors revised the title and explained the issue with negative control for translation-independent mRNA localization. The authors also addressed the issue with interpretation of heat-shock treatment results. The authors have clarified their model in the discussion on the mechanism of erm-1 mRNA and protein localization in polarized cells.

Comments for the author

The authors responded to all my comments and suggestions from the prior review.

© 2022. Published by The Company of Biologists under the terms of the Creative Commons Attribution License (https://creativecommons.org/licenses/by/4.0/).

Minor points:

1. The authors should highlight the prior work on erm-1 mRNA localization (Li et al. Cell Reports 2021) in the introduction and in conjunction with their initial findings. It is referenced later, but that work should be discussed in the introduction, given that the current study is focusing on mechanism of erm-1 mRNA localization and that erm-1 mRNA localization is extensively investigated in that paper.
2. The title in the supplementary materials is the old title.

© 2022. Published by The Company of Biologists under the terms of the Creative Commons Attribution License (https://creativecommons.org/licenses/by/4.0/).
